# Mitochondrial DNA copy number and the risk of autoimmune diseases: A Mendelian randomization study with meta-analysis

**DOI:** 10.1016/j.jtauto.2024.100251

**Published:** 2024-10-02

**Authors:** Mingzhu Liu, Xiongquan Long, Shuangshuang Fu, Yuyang Zhang, Zihao Liu, Xiaoping Xu, Minghao Wu

**Affiliations:** a Department of Gastroenterology, Hunan Provincial People's Hospital, The First Affiliated Hospital of Hunan Normal University, Hunan, 410005, China; b Department of Nephrology and Laboratory of Kidney Disease, Hunan Provincial People's Hospital, The First Affiliated Hospital of Hunan Normal University, Changsha, Hunan, 410005, China; c Department of Endoscopic Diagnosis and Treatment Center, Hunan Cancer Hospital, The Affiliated Cancer Hospital of Xiangya School of Medicine, Central South University, Changsha, Hunan, 410005, China

**Keywords:** Mitochondrial DNA copy number, Autoimmune diseases, Mendelian randomization, Genetic level

## Abstract

**Background:**

Mitochondrial DNA plays a crucial role in the pathophysiology of autoimmune diseases (ADs). However, the association between mitochondrial DNA copy number (mtDNA-CN) and ADs risk is controversial. In this study, Mendelian randomization (MR) analysis and meta-analysis were performed using three sets of independent instrumental variables (IVs) to investigate the potential association between mtDNA-CN and 20 types of ADs.

**Methods:**

The three sets of IVs were drawn primarily from participants in the UK Biobank and the Cohorts for Heart and Aging Research in Genomic Epidemiology consortium using different methods. Outcome data for ADs were investigated using summary statistics from the FinnGen cohort. The potential causal associations were assessed using inverse-variance weighting (IVW), MR-Egger, and weighted median methods. Sensitivity analysis and the Steiger test were used to verify the robustness of the MR estimates. In addition, a meta-analysis was conducted to pool the results from three IV groups.

**Results:**

Overall, genetically predicted mtDNA-CN was not associated with ADs risk (OR = 1.046, 95 % CI: 0.964–1.135, P = 0.283). However, subgroup analyses showed positive causal associations of mtDNA-CN with autoimmune hypothyroidism (OR = 1.133, 95 % CI: 1.016–1.262, P = 0.024) and rheumatoid arthritis (OR = 1.219, 95 % CI: 1.028–1.445, P = 0.023). In contrast, there was no causal association between mtDNA-CN and atopic dermatitis as well as psoriasis, ulcerative colitis, adult-onset Still disease, type1 diabetes, Crohn disease, sarcoidosis, ankylosing spondylitis, multiple sclerosis, autoimmune hyperthyroidism, primary sclerosing cholangitis, systemic lupus erythematosus, systemic sclerosis, alopecia areata, myasthenia gravis, Guillain-Barre syndrome, dermatopolymyositis, and vitiligo.

**Conclusions:**

This MR analysis showed mtDNA-CN is causally associated with an increased risk of autoimmune hypothyroidism and rheumatoid arthritis at the genetic level. The findings have important implications for the use of mtDNA-CN as a biomarker for risk assessment of autoimmune hypothyroidism and rheumatoid arthritis in clinical practice.

## 1 Introduction

Autoimmune diseases (ADs) are chronic diseases in which the immune system responds to self-tissue cells, resulting in cellular injury or tissue damage. ADs affect approximately 5–8% of the world's population and the overall incidence has been steadily increasing [1,2]. However, the pathogenesis of ADs is still not fully elucidated, and it is generally recognized that they are closely related to genetic, environmental, hormonal and immunological factors [3].

Mitochondria are important organelles in human cells and play a crucial role in cellular energy metabolism, cell differentiation, proliferation and aging. Mitochondrial DNA (mtDNA) is the genome of mitochondria itself, encoding 2 ribosomal RNAs, 13 respiratory chain polypeptides, and 22 transfer RNAs [4]. Mitochondrial DNA copy number (mtDNA-CN) reflects the ratio of mitochondrial to nuclear DNA copy number, which is considered to be a surrogate for mitochondrial number and mitochondrial dysfunction, and can indirectly reflect the mtDNA damage [5,6]. Due to the stability of mtDNA and its easy accessibility from the blood, mtDNA-CN has recently attracted interest as a biomarker in diseases. The diagnostic and prognostic value of mtDNA content in different types of ADs was assessed by analyzing tissue specimens from normal and ADs. Several studies found that mtDNA-CN was associated with increased risk of rheumatoid arthritis and systemic lupus erythematosus [7,8]. In contrast, some studies observed that mtDNA-CN was associated with decreased risk of Sjögren's syndrome, multiple sclerosis, and systemic sclerosis [[9], [10], [11]]. Notably, the timing of biological sample collection, either before or after the diagnosis of the disease, may significantly affect the association between mtDNA-CN and ADs. This is because mtDNA-CN fluctuate as the disease progresses. Therefore, we should consider other methods, such as Mendelian randomization (MR), to further validate the association between mtDNA-CN and ADs risk.

Mendelian randomization (MR) analysis is a new and powerful epidemiological tool that has recently gained widespread use in disease etiology studies. MR assesses the causal impact of exposure on outcomes by selecting exposure-related SNPs as instrumental variables (IVs). In detail, this IVs alternative approach mimics the design of randomized controlled trials because SNPs are randomly assigned to offspring at conception, which largely avoids the effects of confounding factors [12,13]. The aim of this study was to determine the causal association between mtDNA-CN and ADs risk by using two-sample MR analysis. The findings will increase our understanding of the role of mtDNA-CN on ADs.

## 2 Materials and methods

### 2.1 MR design

**Fig. 1:**
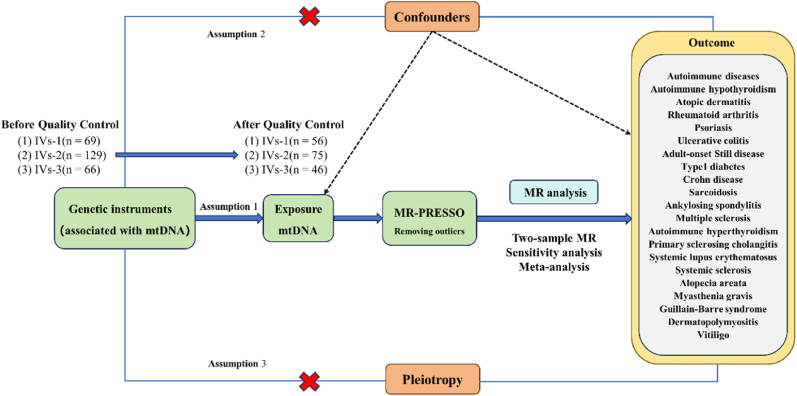
Flowchart of the data collection, processing, and analysis procedures of this study.

We systematically assessed the causal association between mtDNA-CN and ADs by using two-sample MR analysis. Compelling MR analysis should meet 3 basic assumptions: (1) the genetic instruments should be directly associated with exposure (i.e., mtDNA-CN in this study); (2) the genetic instruments should be independent of outcome (i.e., ADs in this study) and independent of any known or unknown confounders; (3) the effect of IVs on outcome is mediated only by the exposure of interest. Genetic information for mtDNA-CN and ADs were obtained from separate genome-wide association studies (GWAS) datasets to avoid sample overlap. The study design was compliant with the STROBE-MR checklist (Table S1) [13]. An overview of this MR study is shown in Fig. 1.

### 2.2 GWAS data for mtDNA-CN and ADs

GWAS summary statistics for mtDNA-CN were derived from three distinct studies: (1) IVs-1 were obtained from Chong et al.'s study [14]. In this study, Chong et al. used a new method called automatic mitochondrial copy (AutoMitoC) to identify genetic factors associated with mtDNA-CN in 395,781 UK Biobank (UKB) participants. The method includes four main steps: preprocessing, background correction, probe cross-hybridization assay, and final mtDNA-CN estimation. AutoMitoC provides a fast, simple, and accurate method for estimating mtDNA concentration in blood samples; (2) IVs-2 were obtained from Longchamps et al.'s study [15]. The above work performed GWAS analyses on 465,809 White individuals from the Cohorts for Heart and Aging Research in the Genomic Epidemiology consortium and UKB; and (3) IVs-3 was extracted from Hägg et al.'s study [16]. This work used the weighted intensities of probes targeting the mitochondrial genomes of 295,150 UKB participants to assess mtDNA content.

To minimize bias due to racial differences and sample overlap, GWAS data for ADs were extracted from the FinnGen cohort on the Integrative Epidemiology Unit (IEU) Open GWAS project (https://gwas.mrcieu.ac.uk/). The FinnGen Biobank GWAS performed by the FinnGen team (https://finngen.gitbook.io/documentation/v/r5/datadownload) involved 42,202 ADs cases and 176,590 genetically matched controls. Characteristics of the GWAS included in the Mendelian randomization were showed in Table S2.

### 2.3 Selection of IVs

In order to fulfill the 3 basic assumptions of the MR study, we identified IVs associated with mtDNA-CN from multiple perspectives through rigorous screening conditions. First, referring to previous studies, we set the significance threshold at p < 5 × 10^−8^ and retain SNPs with linkage disequilibrium (LD) r2 < 0.001 and a long physical distance (window size = 10,000 kilobase) [17,18]. Second, to eliminate the bias caused by poor quality IVs, we calculated the R2 and F-statistics for each SNP (the formulas are shown in Table S3) [19,20]. In general, F-statistics <10 were considered weak IVs and discarded. Third, SNPs with a minor allele frequency (MAF) less than 0.01 were removed. Fourth, we harmonized SNPs for exposure and outcome and eliminated SNPs with palindromic effects and allelic incongruence. Fifth, to avoid the effect of confounders, LDtrait Tool (https://ldlink.nih.gov/?tab=ldtrait) was used to test the assumption that IVs are independent of confounders by analyzing genome-wide significant associations (P < 5 × 10^−8^) [21]. Through this process, we eliminated SNPs associated with smoking and body mass index, two major risk factors for Ads [22,23]. Finally, we used the MR-PRESSO method to identify pleiotropic outliers and removed them before performing MR analysis [24].

### 2.4 MR analysis and statistical analysis

In this MR analysis, the causal association between mtDNA-CN and ADs was primarily estimated using the inverse-variance weighting (IVW) method. It is worth noting that the IVW method allows for a consistent assessment of causality when all three assumptions of a valid instrumental variable are met for each variable [25]. To improve the stability and robustness of the results, we used the MR-Egger and weighted median (WM) methods as supplementary analyses. The MR-Egger method can detect violations of the IVs assumptions and provide estimates of effects that are not affected by these violations [26]. The WM method is more tolerant of invalid IVs and produces plausible estimates when more than half of the weights correspond to valid IVs [27]. In addition, we performed the following sensitivity analyses: (1) Cochran Q test was used to assess the heterogeneity of estimates of IVs. If the Cochran Q test result was P < 0.05, it indicated that there was heterogeneity in the analyzed results [28]; (2) MR-Egger regression and global test of MR-PRESSO was utilized to estimate horizontal pleiotropy, where P > 0.05 indicated no evidence of horizontal pleiotropy [28,29]; (3) we performed leave-one-out analysis to assess whether the results were influenced significantly by a single SNP [30]. Moreover, we used the Steiger test to confirm whether the observed causality was biased due to reversed causation. If Steiger test was P < 0.05, it indicated that causal inference was unbiased [31]. Statistical analyses were performed with R4.3.1 software, and MR analyses were performed with the TwoSampleMR package (version 0.6.2). Additionally, a meta-analysis was performed after pooling the inconsistent results from the three independent IV groups. Meta-analysis was performed using the meta package (version 7.0.0).

## 3 Results

### 3.1 Baseline characteristics of the patients

To investigate the causal association between mtDNA-CN and ADs, three sets of IVs were evaluated. There were 69, 129 and 66 SNPs extracted from the original studies, respectively. After quality control, the number of SNPs for “IVs-1″, “IVs-2″ and “IVs-3″ were 56, 75 and 46, respectively. F-statistical analysis showed that all variables exceeded 10, indicating the absence of potential weak instrumental bias (Table S4). ADs analyses (n = 42202) mainly included 20 types of ADs [autoimmune hypothyroidism (n = 22997), atopic dermatitis (n = 7024), rheumatoid arthritis (n = 6236), psoriasis (n = 4510), ulcerative colitis (n = 4320), adult-onset Still disease (n = 3403), type1 diabetes (n = 2685), Crohn disease (n = 2056), sarcoidosis (n = 2046), ankylosing spondylitis (n = 1462), multiple sclerosis (n = 1048), autoimmune hyperthyroidism (n = 962), primary sclerosing cholangitis (n = 778), systemic lupus erythematosus (n = 538), systemic sclerosis (n = 302), alopecia areata (n = 289), myasthenia gravis (n = 232), multiple sclerosis (n = 213), dermatopolymyositis (n = 208), and vitiligo (n = 131)].

### 3.2 Causal association between mtDNA-CN and ADs based on IVs-1

**Fig. 2:**
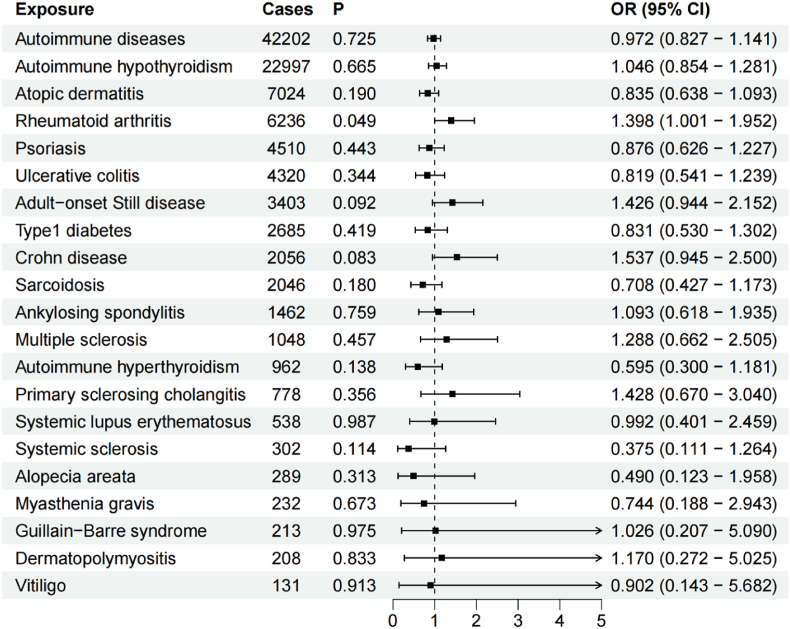
Causal effects of mtDNA-CN on ADs based on IVs-1 by IVW method.

Table S5 presents the results of horizontal pleiotropy, heterogeneity, Steiger test, and MR-PRESSO as well as three MR methods for IVs-1. The IVW method was used as the primary outcome, and the results of the IVW method are shown in Fig. 2.

On the whole, MR analysis indicated that there was no causal association between mtDNA-CN and ADs. In detail, MR analysis revealed the absence of any causal association between mtDNA-CN and autoimmune hypothyroidism as well as atopic dermatitis, psoriasis, ulcerative colitis, adult-onset Still disease, type1 diabetes, Crohn disease, sarcoidosis, ankylosing spondylitis, multiple sclerosis, autoimmune hyperthyroidism, primary sclerosing cholangitis, systemic lupus erythematosus, systemic sclerosis, alopecia areata, myasthenia gravis, Guillain-Barre syndrome, dermatopolymyositis, and vitiligo. On the contrary, mtDNA-CN was positively associated with rheumatoid arthritis (OR = 1.398, 95 % CI: 1.001–1.952, P = 0.049). Moreover, MR-Egger and WM methods had consistent directions with IVW method (OR = 1.516 for the MR-Egger method, OR = 1.310 for the WM method). Cochran Q test showed no heterogeneity. MR-Egger regression and global test of MR-PRESSO suggested that there was no pleiotropy. Additionally, Steiger test indicated that there was no reverse causality effect.

Fig. S1 presents the results of the leave-one-out sensitivity analysis. The results indicated that the association between mtDNA-CN and autoimmune hypothyroidism was influenced by rs11085147 and rs5759176. The association between mtDNA-CN and ankylosing spondylitis was influenced by rs6045561. Furthermore, the association between mtDNA-CN and dermatopolymyositis was influenced by rs11553699, rs12247015, rs74874677, and rs1760940. However, removal of these SNPs separately did not affect the causal associations (Table S6).

### 3.3 Causal association between mtDNA-CN and ADs based on IVs-2

**Fig. 3:**
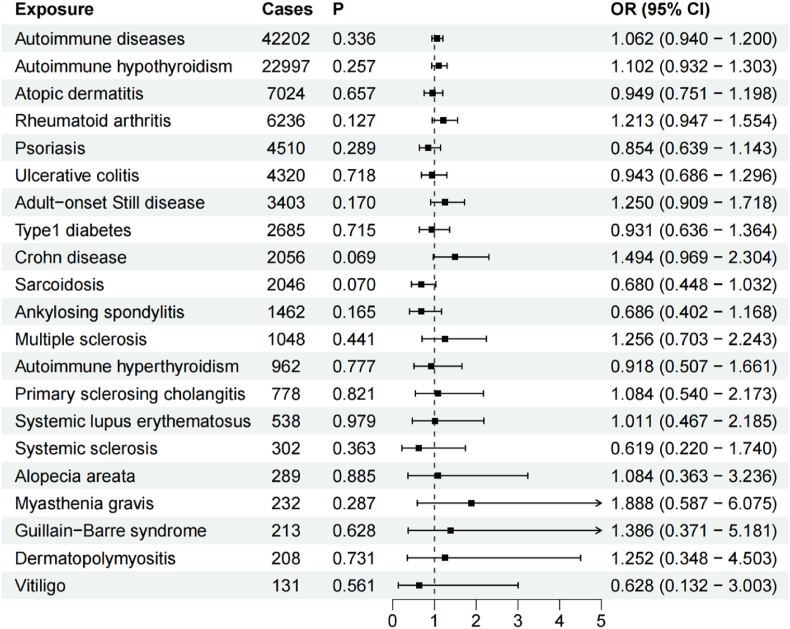
Causal effects of mtDNA-CN on ADs based on IVs-2 by IVW method.

Table S7 summarizes the results of MR-PRESSO, horizontal pleiotropy, and heterogeneity analyses as well as three MR methods for IVs-2. The IVW method was used as the primary outcome, and the results of the IVW method are presented in Fig. 3.

MR analysis revealed no causal association between mtDNA-CN and ADs. Fig. S2 presents the results of the leave-one-out sensitivity analysis. There were no meaningful causal associations after separately removing SNPs that might influence the MR results (Table S6).

### 3.4 Causal association between mtDNA-CN and ADs based on IVs-3

**Fig. 4:**
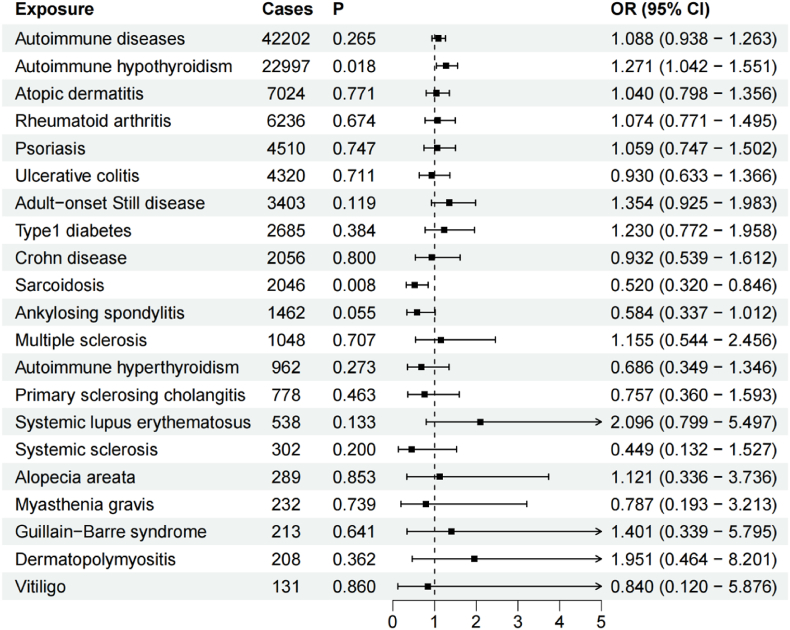
Causal effects of mtDNA-CN on ADs based on IVs-3 by IVW method.

Table S8 presents the results of horizontal pleiotropy, heterogeneity, Steiger test, and MR-PRESSO as well as three MR methods for IVs-3. The IVW method was used as the primary outcome, and the results of the IVW method are shown in Fig. 4.

Overall, MR analysis indicated that there was no causal association between mtDNA-CN and ADs. Specifically, MR analysis revealed the absence of any causal association between mtDNA-CN and atopic dermatitis as well as rheumatoid arthritis, psoriasis, ulcerative colitis, adult-onset Still disease, type1 diabetes, Crohn disease, ankylosing spondylitis, multiple sclerosis, autoimmune hyperthyroidism, primary sclerosing cholangitis, systemic lupus erythematosus, systemic sclerosis, alopecia areata, myasthenia gravis, Guillain-Barre syndrome, dermatopolymyositis, and vitiligo. In contrast, mtDNA-CN was associated with autoimmune hypothyroidism (OR = 1.271, 95 % CI: 1.042–1.551, P = 0.018) and sarcoidosis (OR = 0.520, 95 % CI: 0.320–0.846, P = 0.008). Notably, the causal association between mtDNA-CN and sarcoidosis was unreliable because the MR-Egger method (OR = 1.480) was not consistent with the direction for the IVW method. Between mtDNA-CN and autoimmune hypothyroidism, MR-Egger and WM methods had consistent directions with IVW method (OR = 1.459 for the MR-Egger method, OR = 1.166 for the WM method). Cochran Q test showed no heterogeneity. MR-Egger regression and global test of MR-PRESSO suggested that there was no pleiotropy. Moreover, Steiger test indicated that there was no reverse causality effect.

Fig. S3 presents the results of the leave-one-out sensitivity analysis. The results indicated that the association between mtDNA-CN and alopecia areata was influenced by rs806709 and rs210143. The association between mtDNA-CN and atopic dermatitis, multiple sclerosis, myasthenia gravis, psoriasis, and ulcerative colitis was influenced by rs75107793, rs806709, rs59488041, rs4895441, and rs806709, respectively. However, removal of these SNPs separately did not affect the causal associations (Supplementary Table 6).

### 3.5 Meta-analysis of the MR results of the three IV groups

**Fig. 5:**
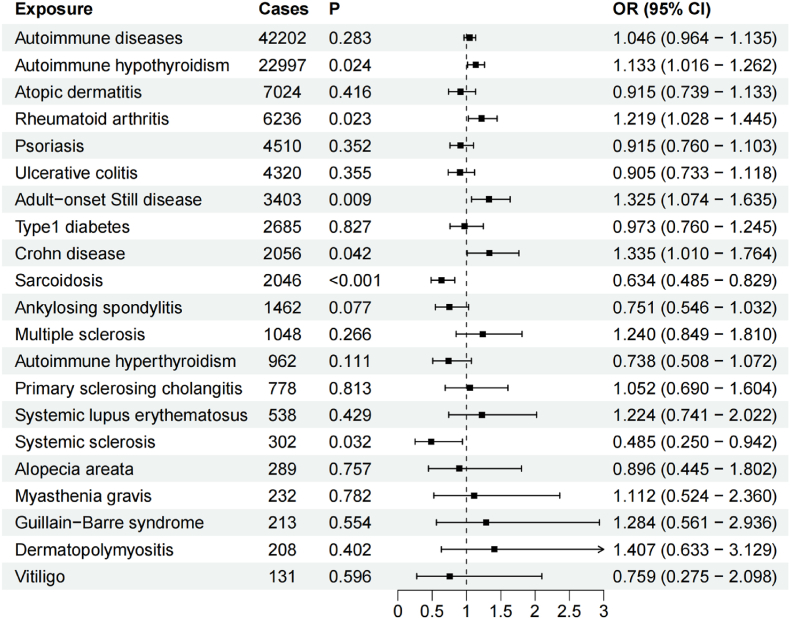
Causal effects of mtDNA-CN on ADs based on meta-analysis by IVW method.

Owing to inconsistent results from the MR analyses of the three IV groups, a meta-analysis was conducted (Fig. 5 and Table S9). On the whole, the results indicated that there was no causal association between mtDNA-CN and ADs. Concretely, the results indicated that there was no causal association between mtDNA-CN and atopic dermatitis as well as psoriasis, ulcerative colitis, type1 diabetes, ankylosing spondylitis, multiple sclerosis, autoimmune hyperthyroidism, primary sclerosing cholangitis, systemic lupus erythematosus, alopecia areata, myasthenia gravis, Guillain-Barre syndrome, dermatopolymyositis, and vitiligo. In contrast, we found that mtDNA-CN was associated with Crohn disease (pooled OR = 1.335, 95 % CI: 1.010–1.764, P = 0.042), adult-onset Still disease (pooled OR = 1.325, 95 % CI: 1.074–1.635, P = 0.009) and systemic sclerosis (pooled OR = 0.485, 95 % CI: 0.250–0.942, P = 0.032). However, despite the statistical significance of the pooled result, all three estimates included in the meta-analysis were negative. These results should be interpreted cautiously. Moreover, the causal association between mtDNA-CN and sarcoidosis (pooled OR = 0.634, 95 % CI: 0.485–0.829, P = 9.0x10^−4^) was not robust because the MR-Egger method (OR = 1.207) was not consistent with the direction for the IVW method. Finally, we found that mtDNA-CN was positively associated with the risk of autoimmune hypothyroidism (pooled OR = 1.133, 95 % CI: 1.016–1.262, P = 0.024) and rheumatoid arthritis (pooled OR = 1.219, 95 % CI: 1.028–1.445, P = 0.023). These two causal associations were reliable because a large number of patients were included and MR-Egger and WM methods had consistent directions with IVW method. Moreover, the meta-analysis showed low SNP heterogeneity, which reduced the introduction of bias.

## 4 Discussion

To our knowledge, this is the first study to use two-sample MR analysis and meta-analysis to comprehensively assess the causal role of mtDNA-CN on ADs. Overall, our findings suggest mtDNA-CN was not associated with ADs risk. However, subgroup analyses showed positive causal associations of mtDNA-CN with autoimmune hypothyroidism and rheumatoid arthritis. In contrast, the results indicate that there was no causal association between mtDNA-CN and atopic dermatitis as well as psoriasis, ulcerative colitis, adult-onset Still disease, type1 diabetes, Crohn disease, sarcoidosis, ankylosing spondylitis, multiple sclerosis, autoimmune hyperthyroidism, primary sclerosing cholangitis, systemic lupus erythematosus, systemic sclerosis, alopecia areata, myasthenia gravis, Guillain-Barre syndrome, dermatopolymyositis, and vitiligo.

In recent years, more and more attention has been paid to the role of mitochondria on ADs, especially the influence of mtDNA on the pathogenesis of ADs. Several studies have shown that alterations in mtDNA can lead to the occurrence of ADs [[32], [33], [34]]. One of the classic views is that various cellular stress conditions trigger the release of mtDNA from the mitochondria into the cytoplasm to induce expression of type I interferon and other effector genes, leading to ADs [35]. Moreover, the production of type I interferon increases the formation of neutrophil extracellular traps enriched in oxidized mtDNA, further aggravating ADs [36,37]. In addition, mtDNA mutations can affect cellular energy metabolism and may contribute to ADs [38,39]. Due to the stability of mtDNA and its easy accessibility through the bloodstream, researchers developed mtDNA-CN to reflect the health of mitochondria. It is important to note that there is no single interpretation of mtDNA-CN, and either a decrease or increase in mtDNA-CN can indicate dysfunction. The mtDNA-CN has been recognized as a novel biomarker for the risk assessment of ADs.

Rheumatoid arthritis is a chronic autoimmune disease of complex etiology characterized by synovial cell hyperplasia, inflammatory cell mass production and cartilage destruction. Studies have shown that mitochondrial dysfunction plays an important contributory role in the development of rheumatoid arthritis. The current view is that there are three main pathways by which mitochondrial dysfunction leads to rheumatoid arthritis, including abnormal energy metabolism, overproduction of reactive oxygen species, and innate immune activation [40,41]. Lehmann et al. conducted a prospective study in a Swiss population and found that plasma mtDNA-CN was increased 19.4-fold in patients with rheumatoid arthritis and positively correlated with disease activity [8]. This is consistent with our findings. However, a study by Svendsen et al. showed that mtDNA-CN of white blood cell is decreased in rheumatoid arthritis and linked with risk factors [42]. The discrepancy in findings may be due to differences in mtDNA-CN data sources and mtDNA-CN measurement methods. The differential content of mtDNA-CN in different cells or tissues in rheumatoid arthritis and its potential mechanisms affecting the development of rheumatoid arthritis need to be further investigated. Autoimmune hypothyroidism is one of the most common thyroid disorders caused by Hashimoto's thyroiditis. Our study showed that mtDNA-CN was positively associated with the risk of autoimmune hypothyroidism. Regrettably, there is no study on the association between mitochondrial functions and the risk of autoimmune hypothyroidism.

Giaglis et al. showed that mtDNA-CN was significantly increased in plasma in patients with systemic lupus erythematosus and was an independent marker representing the activity of systemic lupus erythematosus [7]. Moreover, Movassaghi et al. showed that mtDNA-CN was significantly reduced and the degree of mtDNA damage was exacerbated in patients with systemic sclerosis [9]. Al-Kafaji et al. found that a decrease in peripheral blood mtDNA-CN may be an early symptom of multiple sclerosis and is associated with disease progression [10]. In addition, Vaseghi et al. showed that blood mtDNA-CN and mtDNA damage were higher in vitiligo patients than in healthy controls, which may be related to the pathogenesis of vitiligo [43]. However, our findings did not show a causal association between mtDNA-CN and systemic lupus erythematosus, systemic sclerosis, multiple sclerosis, and vitiligo. Currently, prospective and basic studies on the association between mtDNA-CN and the risk of ADs are still very limited. Our study provides a new comprehensive understanding of the role of mtDNA-CN on ADs risk.

In order to comprehensively analyze the causal association between mtDNA-CN and ADs risk, it is necessary to assess mtDNA-CN in a precise and meticulous manner in order to eliminate any potential bias that may lead to ambiguous results. Therefore, we used three independent groups of IVs, each with different assessment methods, to minimize potential bias. Chong et al. developed AutoMitoC, a method for estimating mtDNA-CN in gene array data to identify common and rare genetic variants [14]. Longchamps et al. performed a GWAS study to identify new SNPs with statistically significant effects on mtDNA-CN [15]. Hägg et al. identified specific SNPs that were significantly correlated with mtDNA abundance [16]. To minimize possible bias due to differences in mtDNA assessment methods and participants, this study pooled the inconsistent results from the three IV groups in a meta-analysis.

This study has some strengths. First, this study provides a comprehensive MR analysis of the causal association between mtDNA-CN and ADs based on a large-scale global genome study dataset, which allows us to better understand the role of mtDNA-CN on ADs. Second, we employed rigorous MR analysis to discard the inevitable pitfalls of previous studies, such as confounding interference and reverse causality. Specifically, the consistency of the three MR method estimates in direction and magnitude ensured the robustness of results. Moreover, sensitivity analyses and Steiger tests were performed to rule out the effects of heterogeneity, pleiotropy, and reverse causality on the estimates. Finally, a meta-analysis was conducted to reduce potential bias.

However, there are some limitations to our study. First, our study is based mainly on European populations. Although this largely avoids population heterogeneity, MR results should be further validated in other populations to verify their generalizability. Second, the sample size of patients with certain types of ADs in the FinnGen dataset is small. A large sample size is required to validate the results. Third, although this study excluded IVs associated with smoking and body mass index, it was a limitation that many other ADs risk factors could not be fully accounted for. Finally, due to the limitation of the number of mtDNA-CN-associated SNPs, we were unable to perform a comprehensive reverse MR analysis.

## 5 Conclusion

In summary, the results of our analyses suggest that mtDNA-CN is causally associated with an increased risk of autoimmune hypothyroidism and rheumatoid arthritis at the genetic level. This reminds that people with elevated blood mtDNA-CN concentrations need to pay attention to early autoimmune hypothyroidism and rheumatoid arthritis screening. More observational and basic studies are required to further understand and validate the association between mtDNA-CN and ADs.

## Funding

This work was supported by the 10.13039/501100001809National Natural Science Foundation of China (Grant Number: 81900466), the Natural Science Foundation Youth Project of Hunan Provincial (Grant Number: 2022JJ40217), the Hunan Provincial Health Commission Project (Grant Number: 202203053635), and the Excellent Youth Fund Project of Hunan Provincial Education Department (Grant Number: 22B0061).

## Ethics statement

This MR research used only published or publicly available GWAS data. Each participant received ethical approval and informed consent for the respective study, as detailed in the original publication and consortium.

## CRediT authorship contribution statement

**Mingzhu Liu:** Writing – review & editing, Writing – original draft, Methodology, Formal analysis, Data curation. **Xiongquan Long:** Writing – original draft, Methodology, Formal analysis, Conceptualization. **Shuangshuang Fu:** Funding acquisition, Data curation, Conceptualization. **Yuyang Zhang:** Visualization, Validation, Investigation. **Zihao Liu:** Validation. **Xiaoping Xu:** Writing – review & editing, Supervision, Project administration, Funding acquisition, Conceptualization. **Minghao Wu:** Writing – review & editing, Supervision, Project administration, Investigation, Conceptualization.

## Declaration of competing interest

The authors declare that they have no known competing financial interests or personal relationships that could have appeared to influence the work reported in this paper.

## Data Availability

Only publicly available data were used in this study, and data sources and handling of these data are described in the Materials and Methods and in Table S2.
