# Supplementary material for: Mitochondrial DNA copy number and the risk of autoimmune diseases: A Mendelian randomization study with meta-analysis
Source: J Transl Autoimmun. 2024 Oct 2;9:100251. doi: 10.1016/j.jtauto.2024.100251 (PMC11491893; doi:10.1016/j.jtauto.2024.100251)
Supplement: Multimedia component 1 [file mmc1.docx]

**Mitochondrial DNA copy number and autoimmune diseases: a Mendelian randomization study with meta-analysis**

**Supplementary Figures**

**Supplementary Figure 1.** Forest plots for the Mendelian randomization (MR) leave-one-out analysis based on IVs-1.

**Supplementary Figure 2.** Forest plots for the Mendelian randomization (MR) leave-one-out analysis based on IVs-2.

**Supplementary Figure 3.** Forest plots for the Mendelian randomization (MR) leave-one-out analysis based on IVs-3.


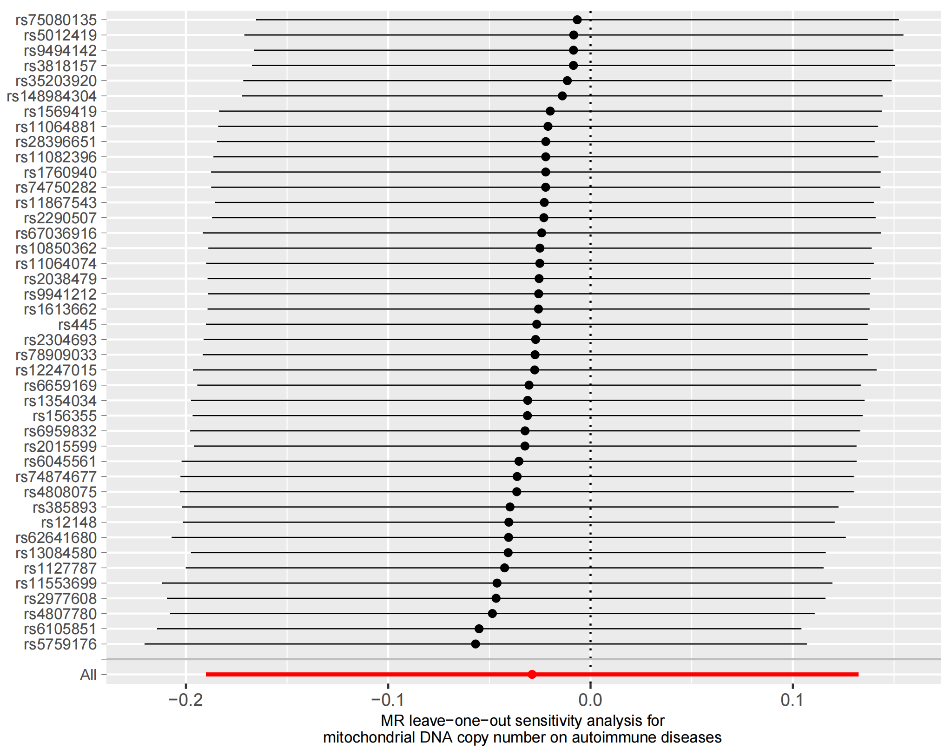

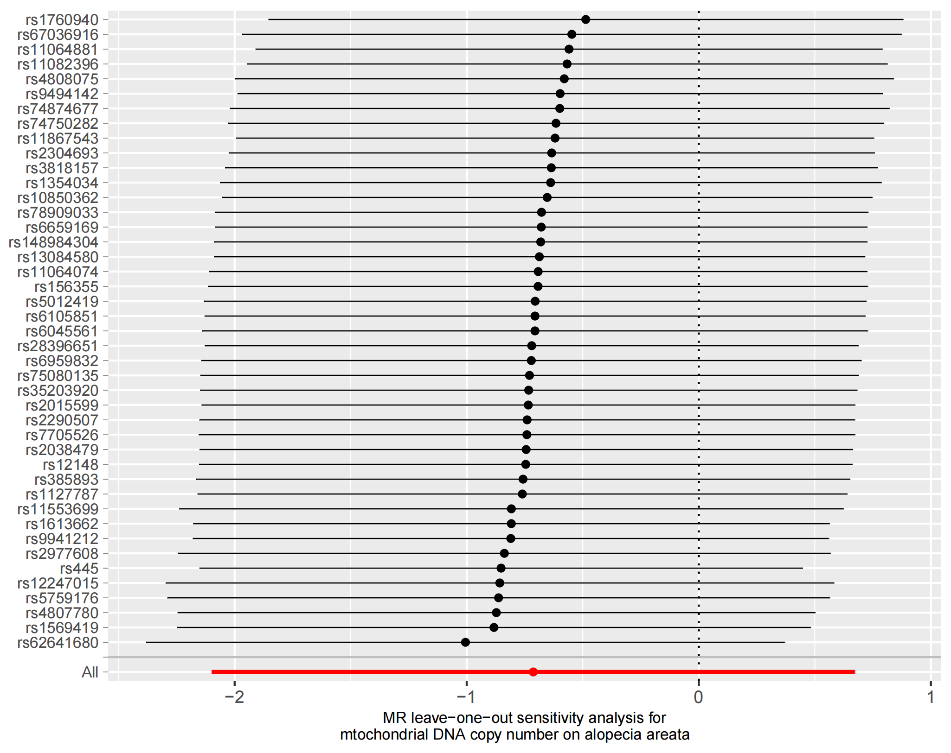


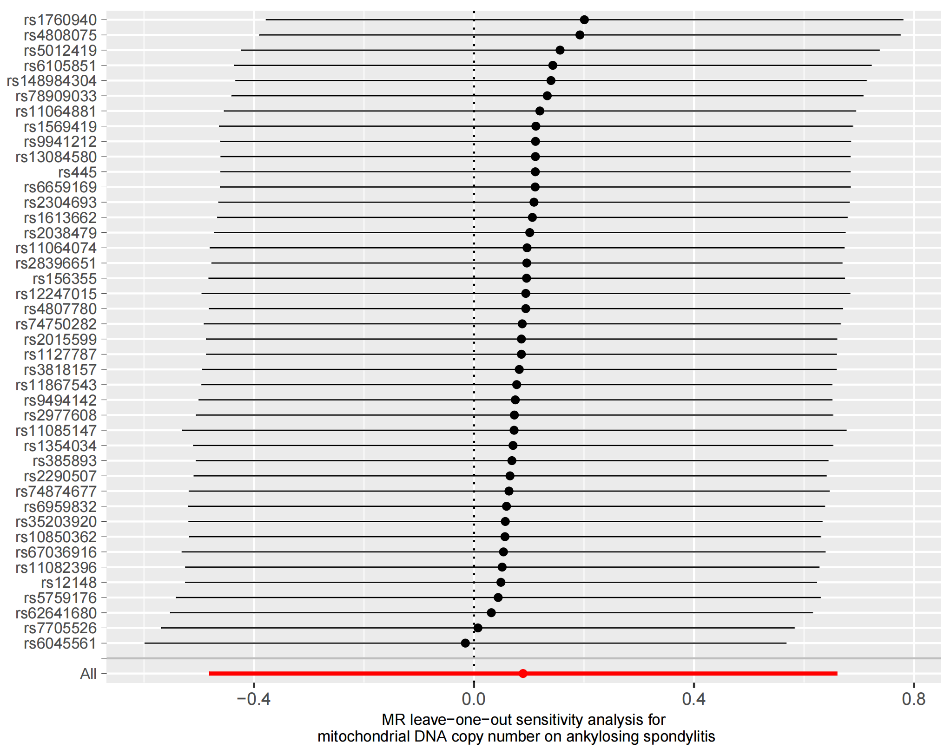

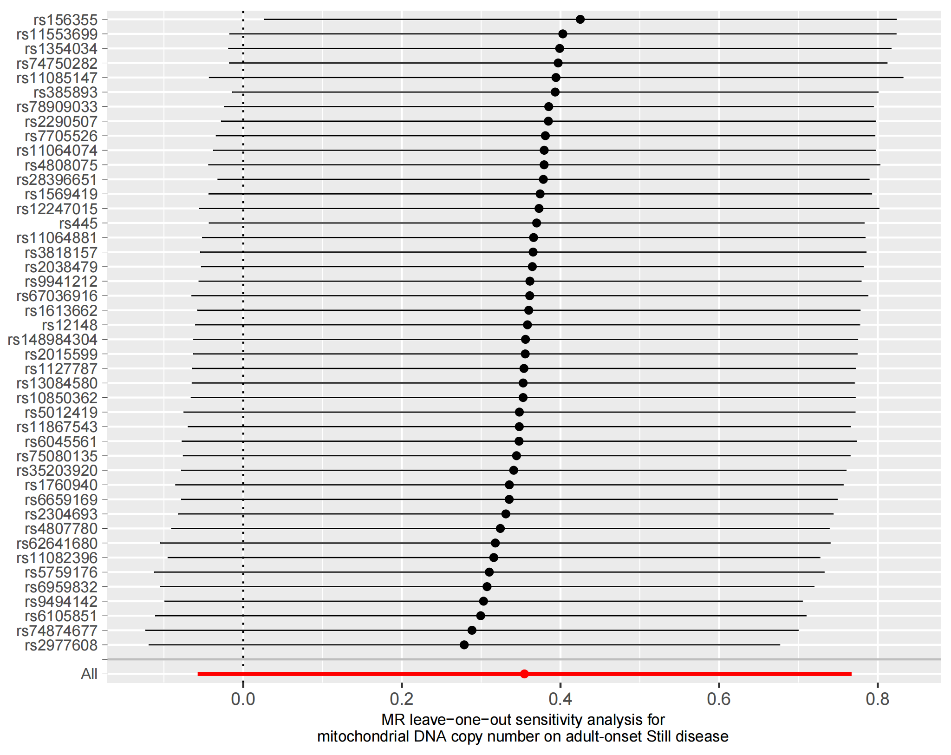


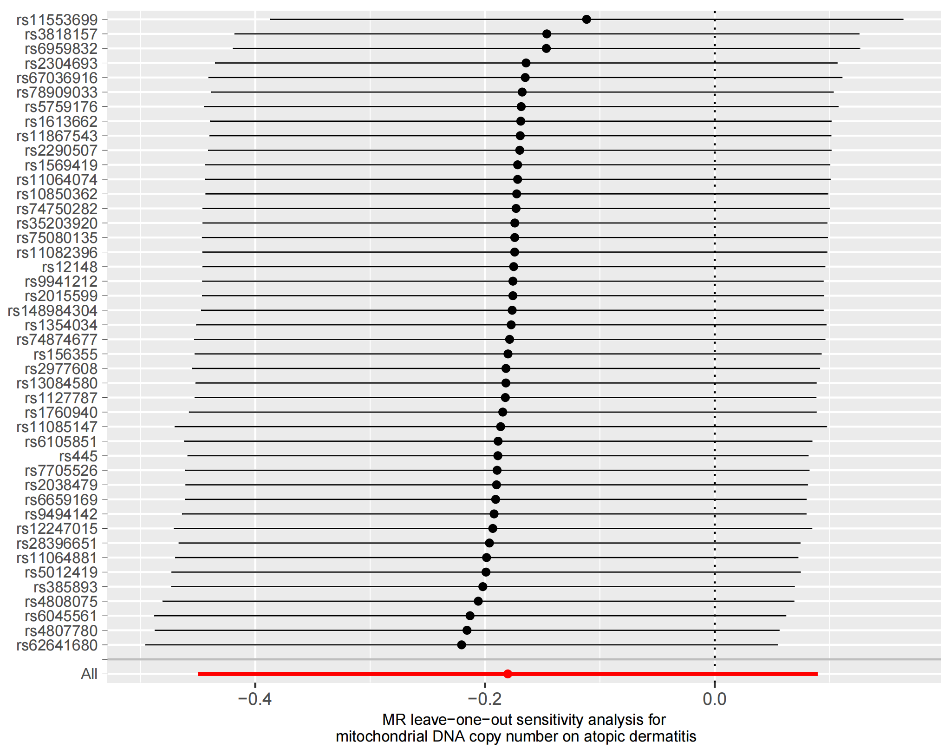

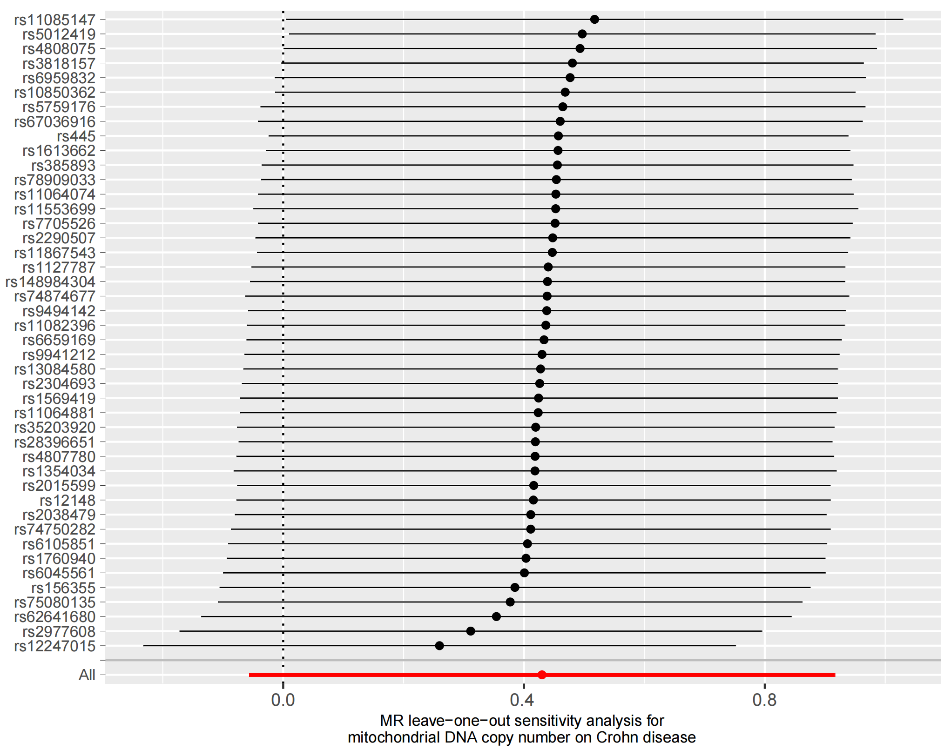


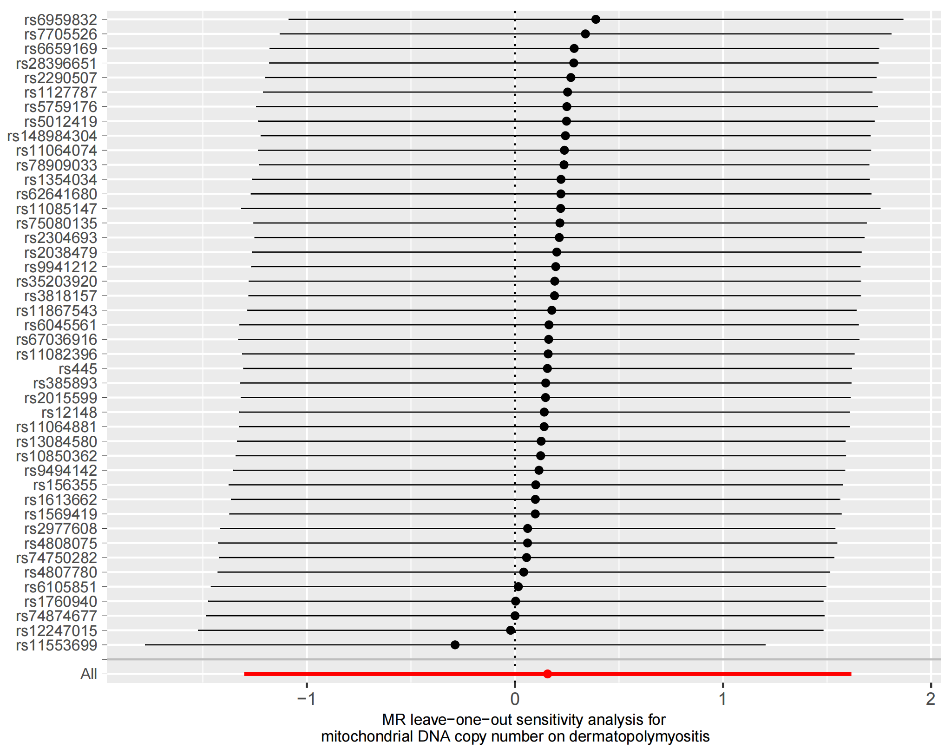

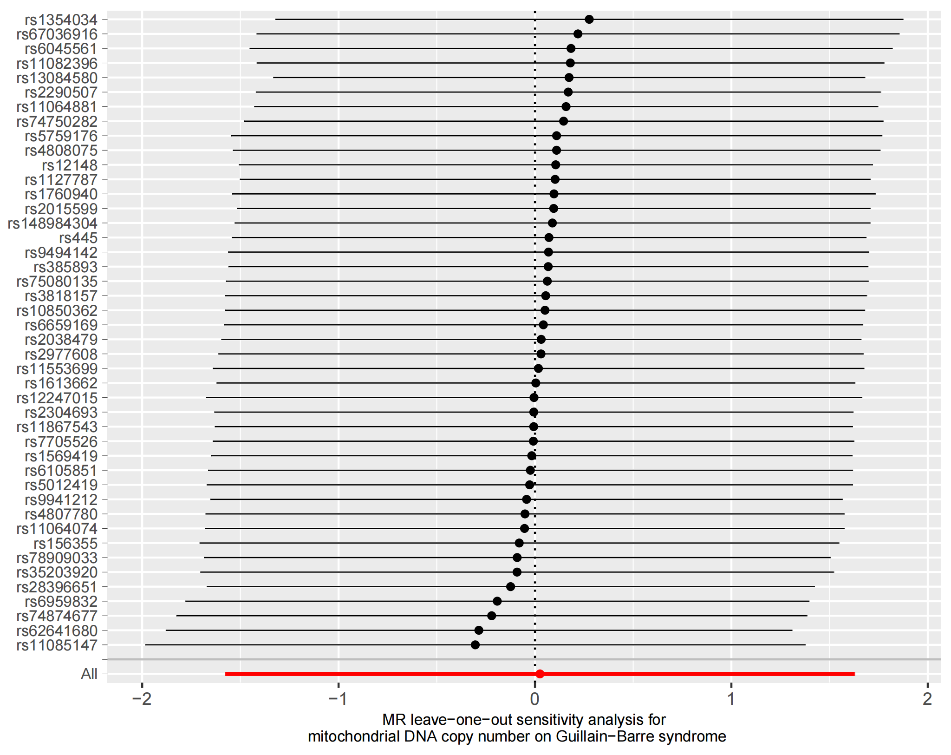


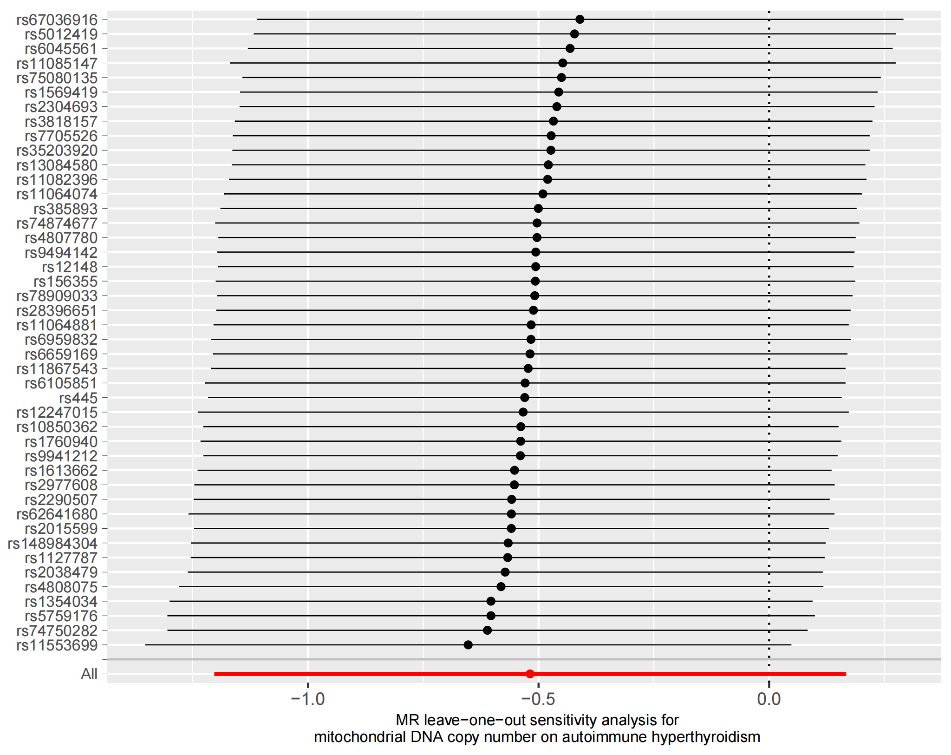

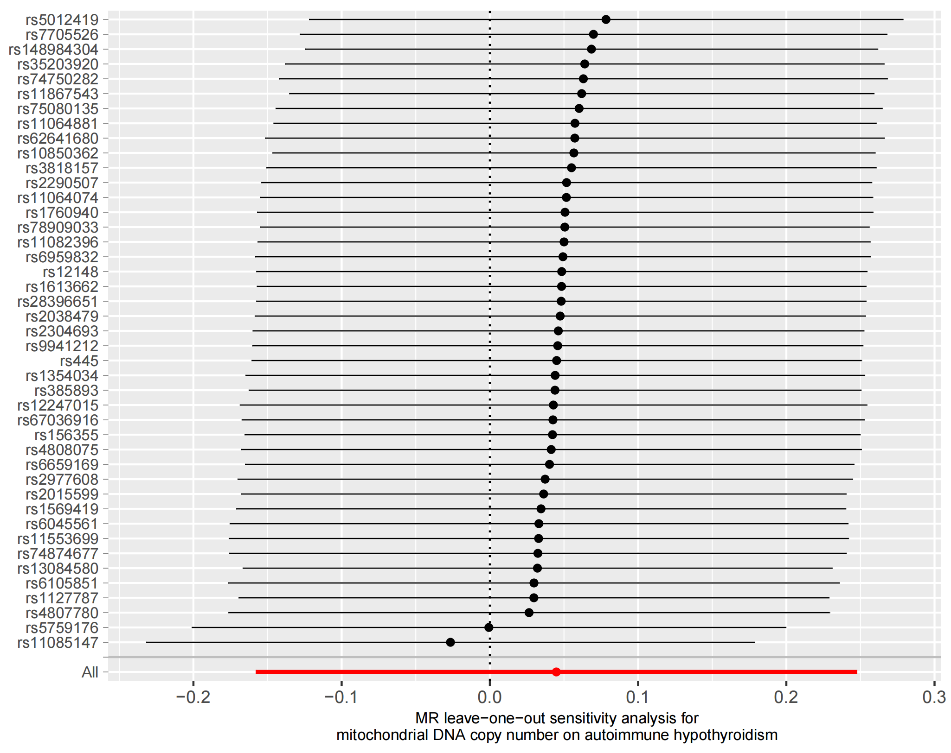


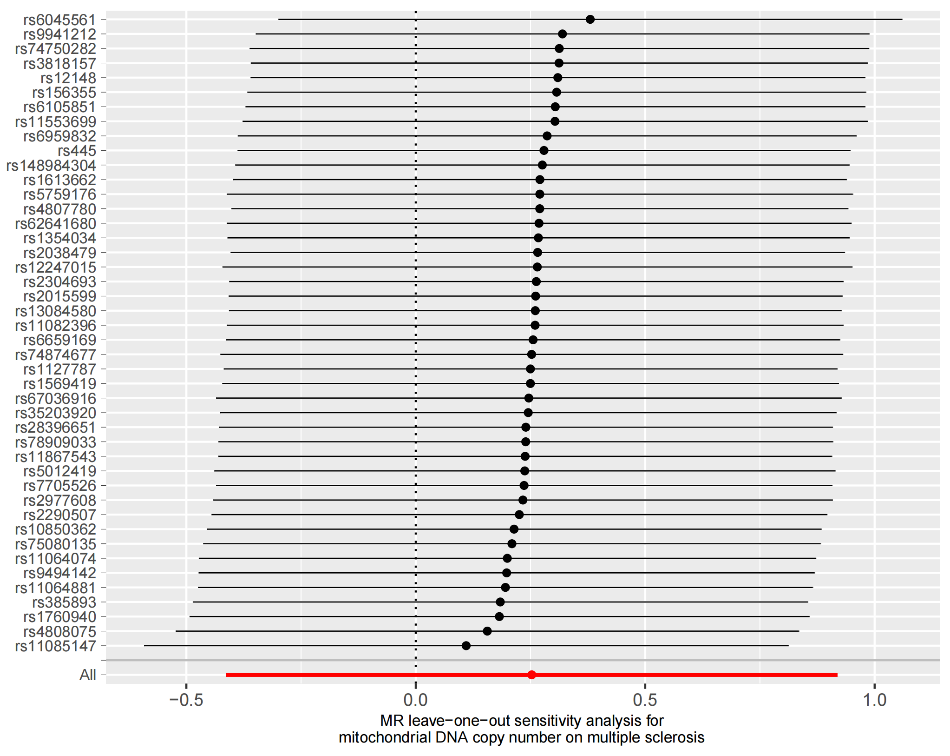

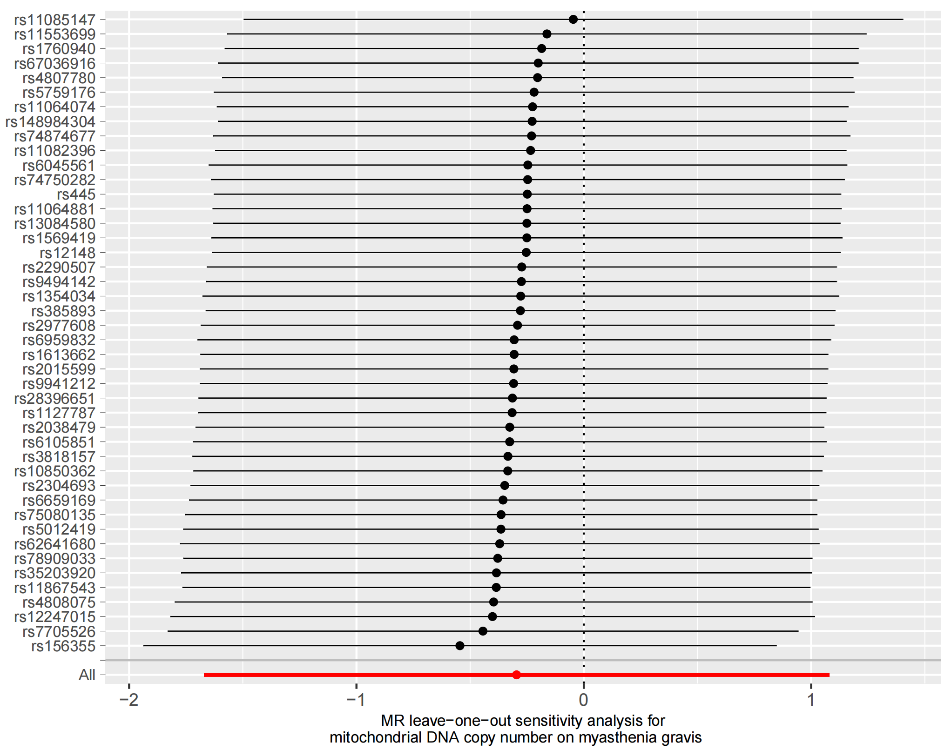


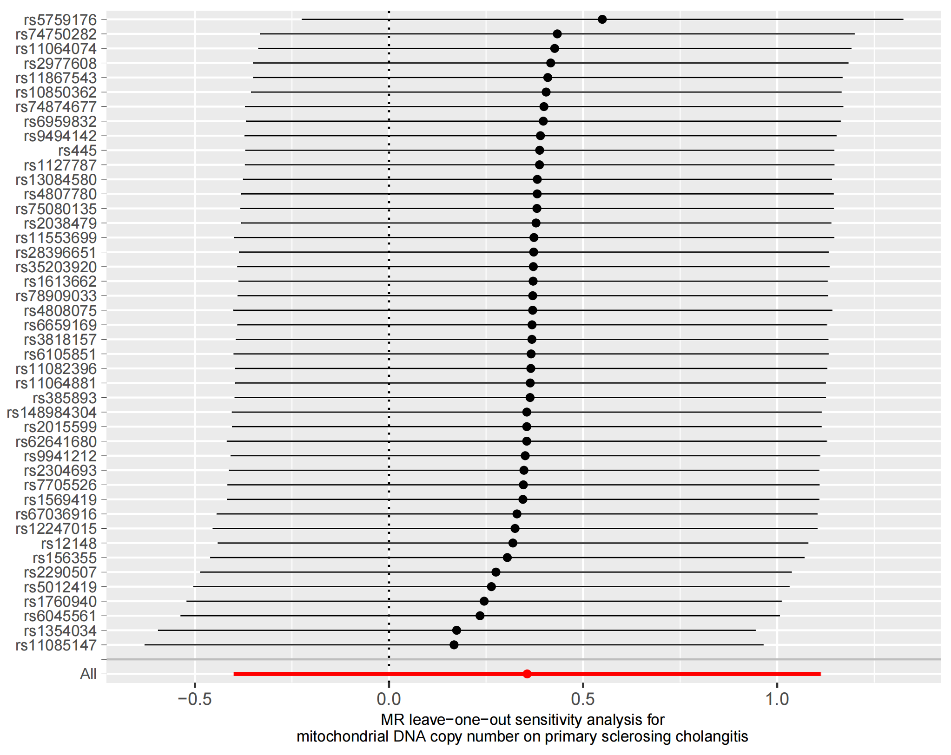

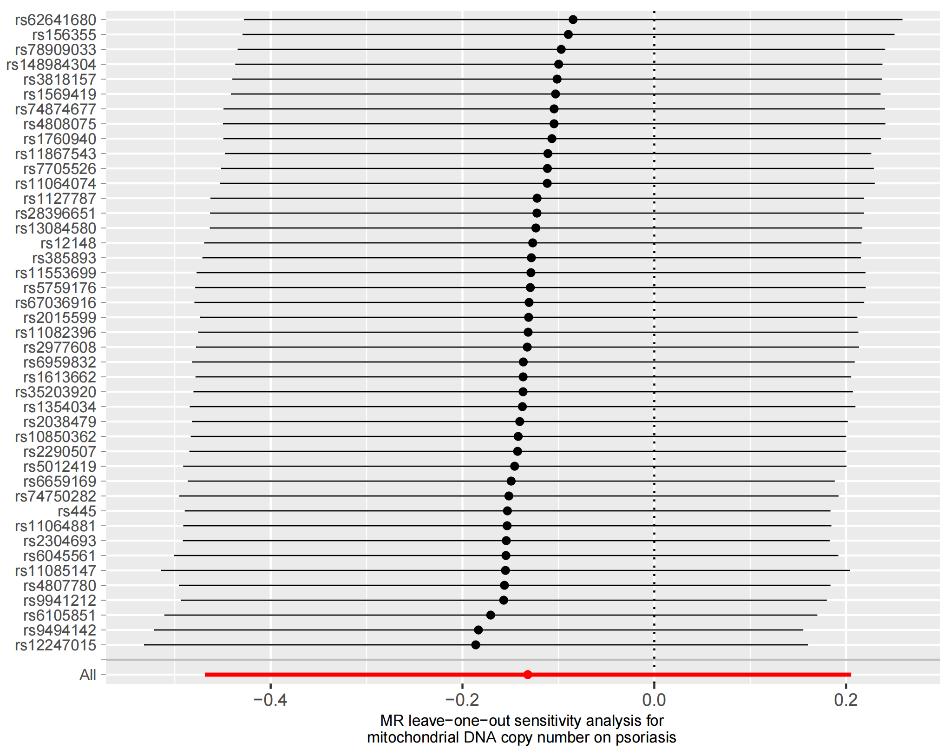


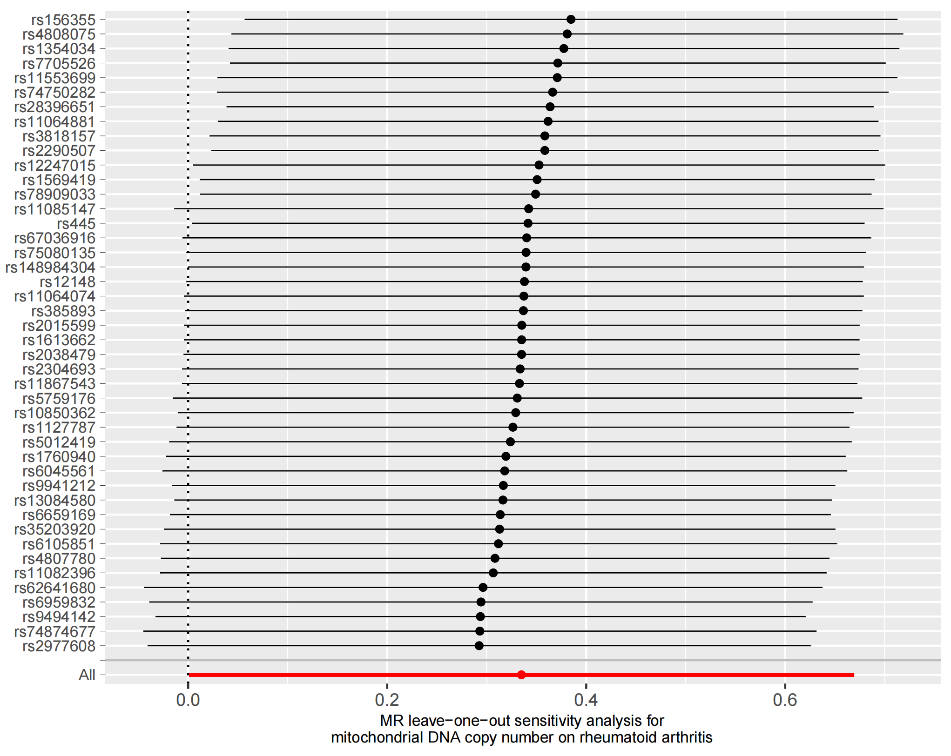

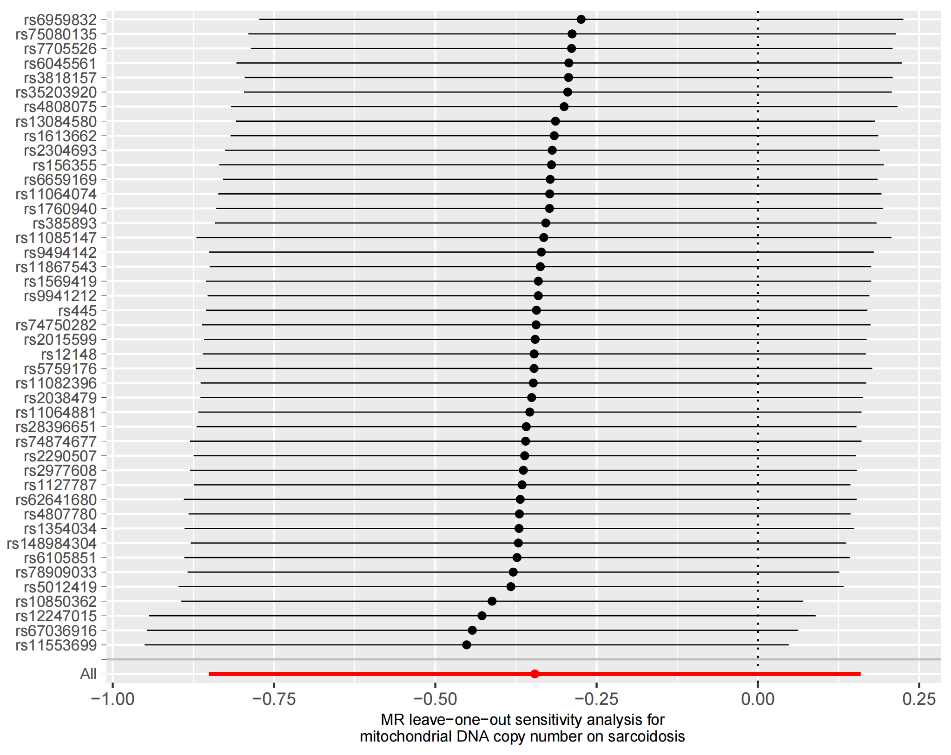


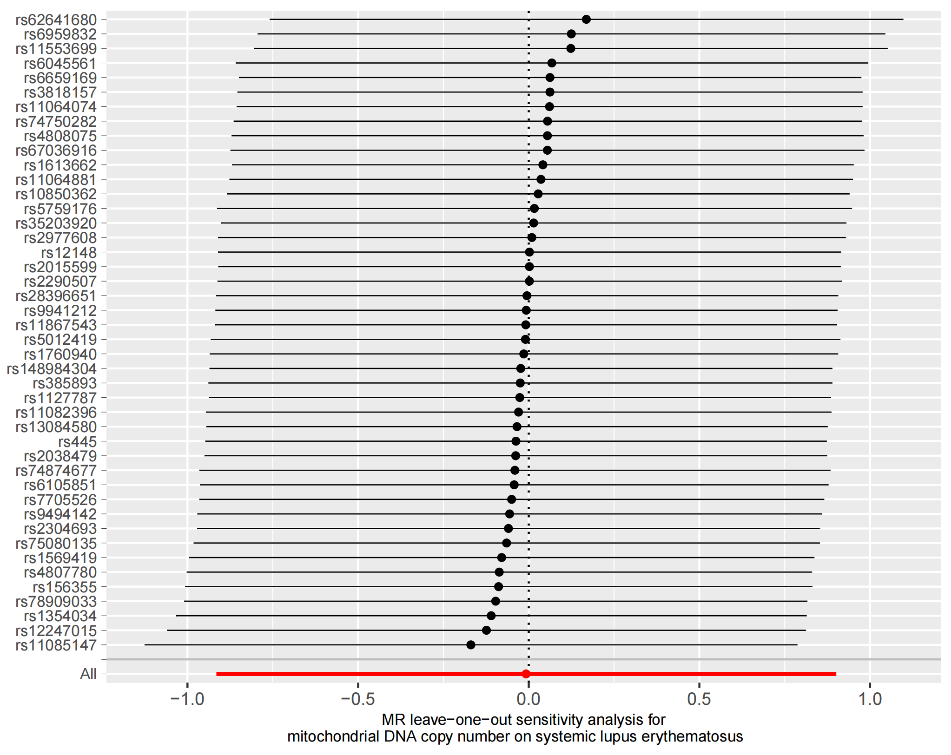

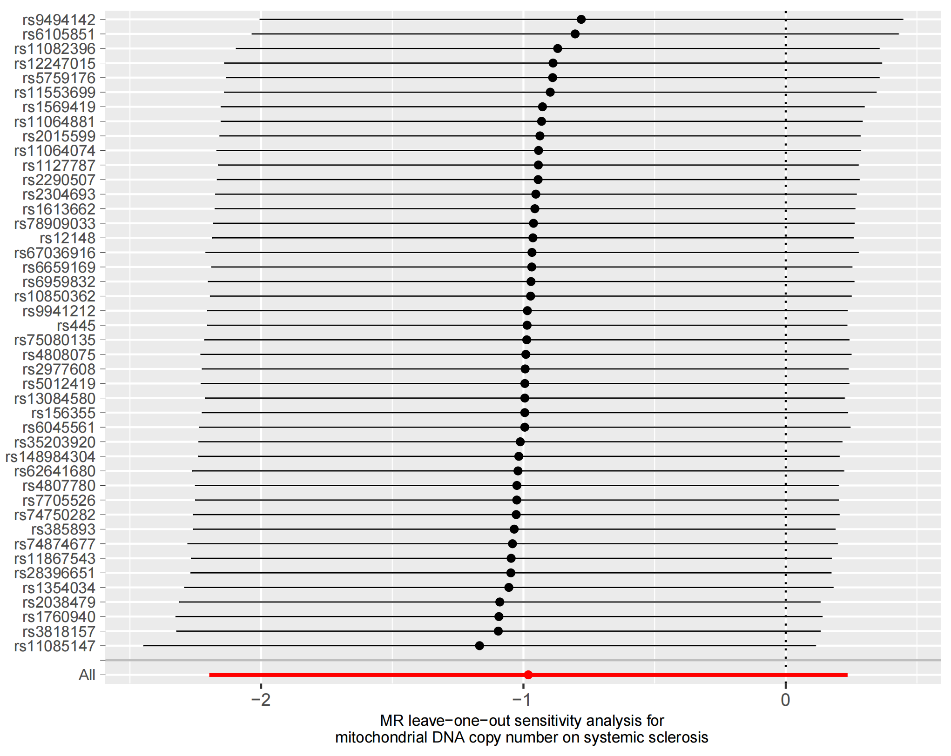


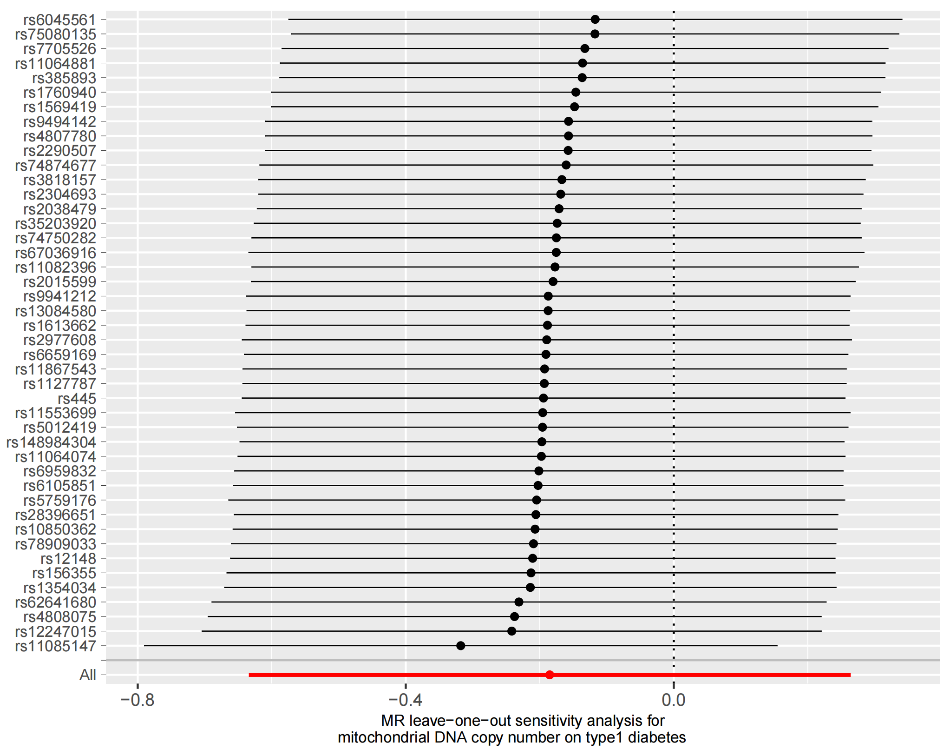

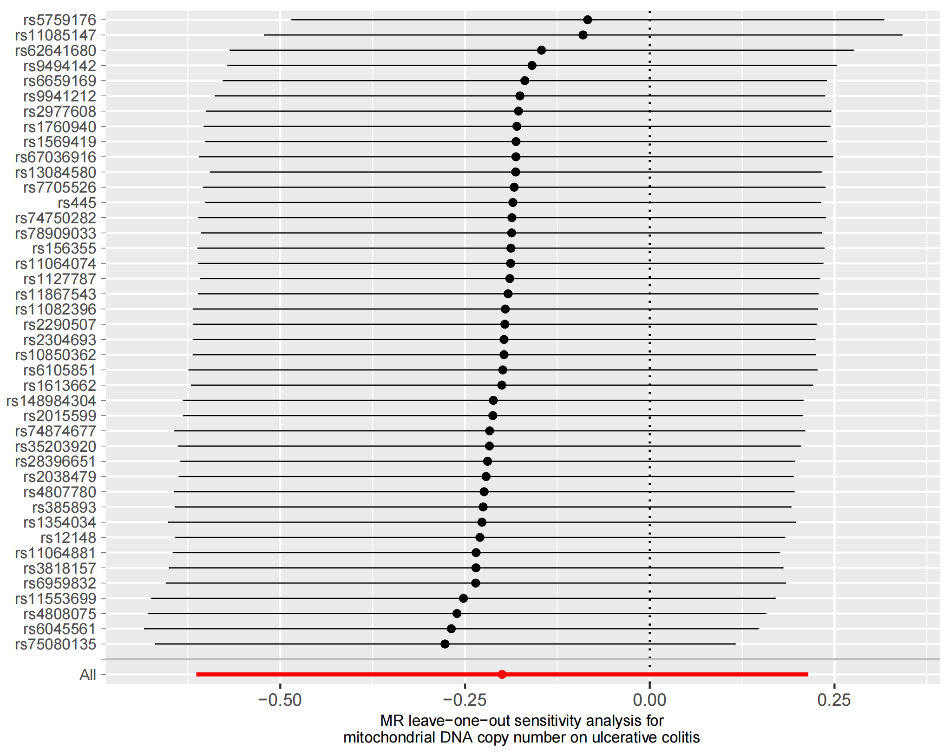


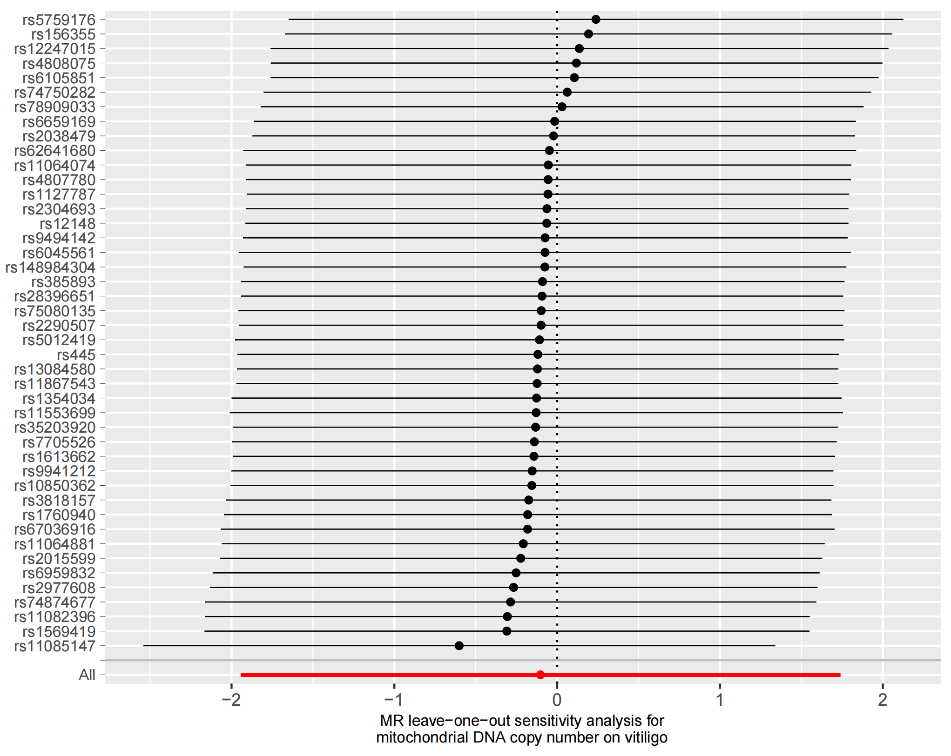


**Supplementary Figure 1.** Forest plots for the Mendelian randomization (MR) leave-one-out analysis based on IVs-1.


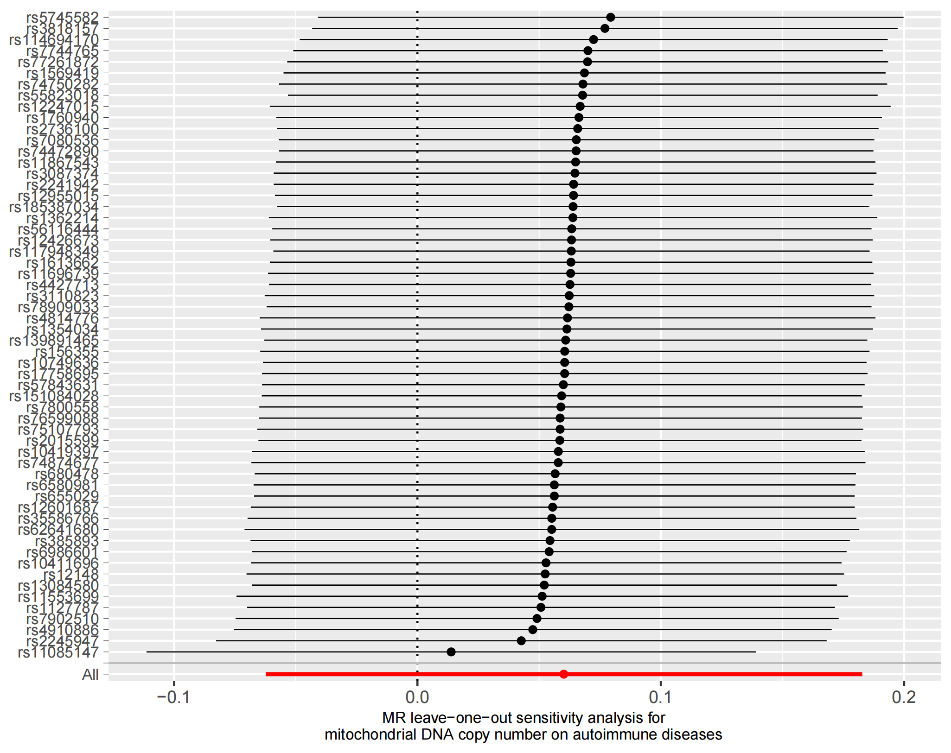

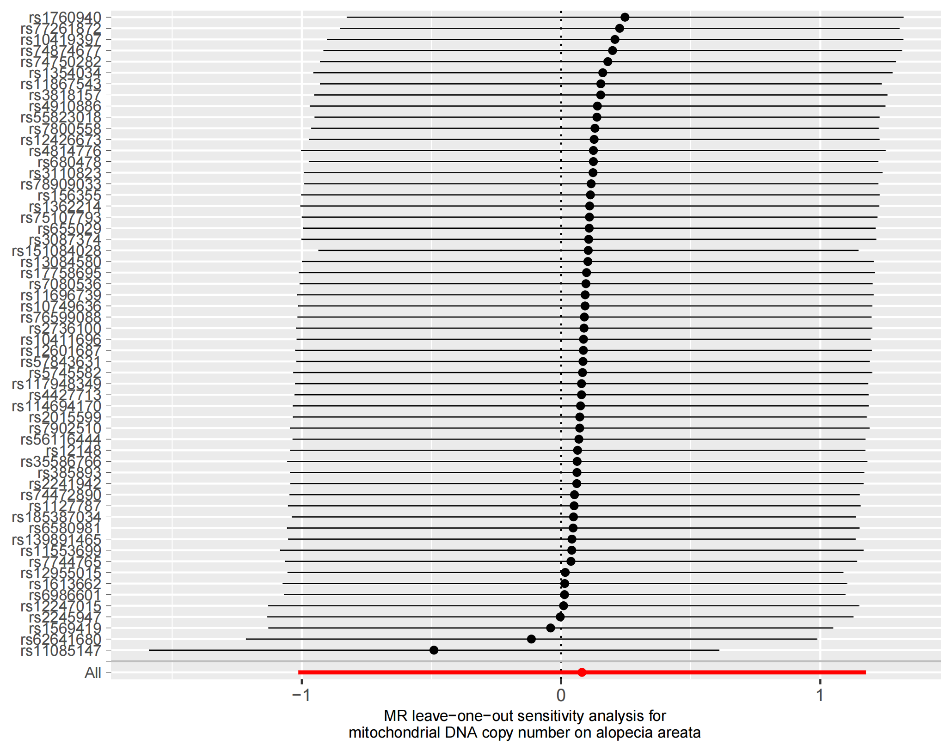


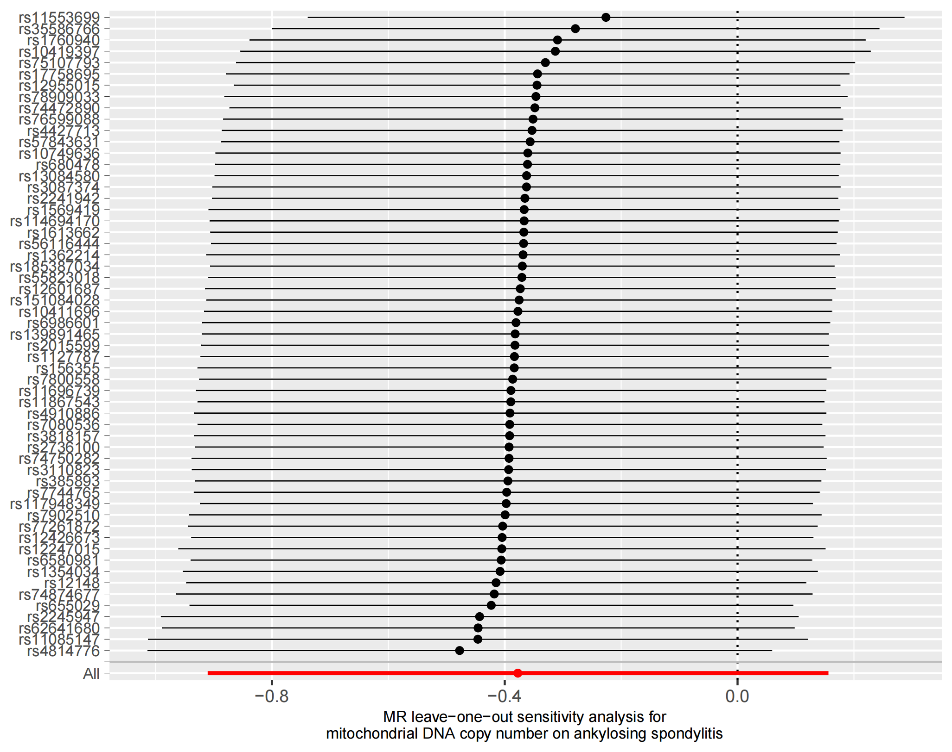

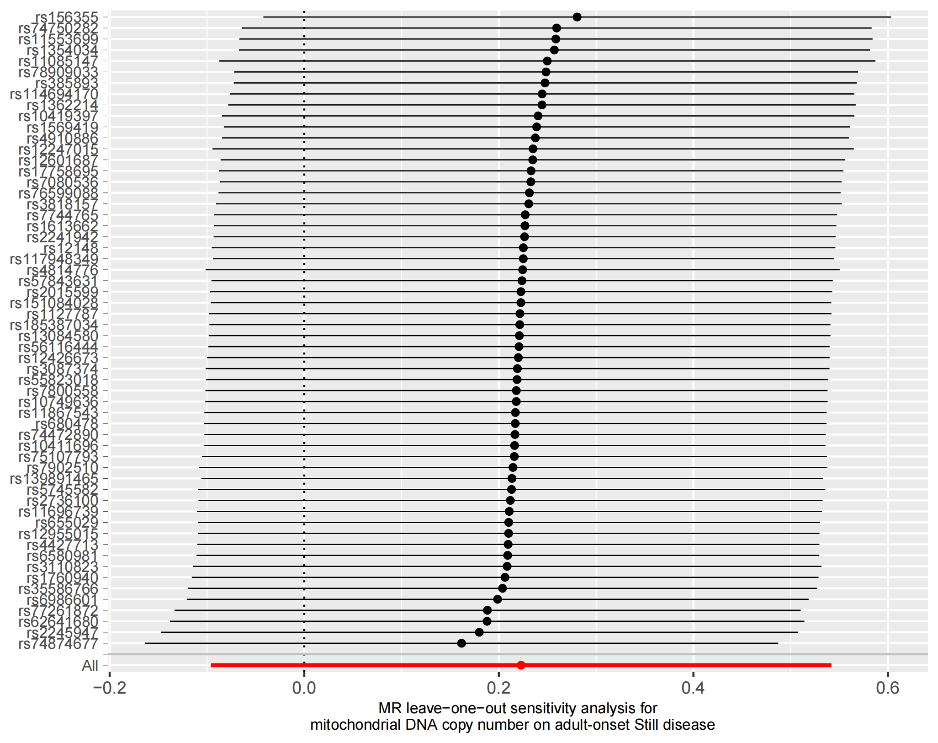


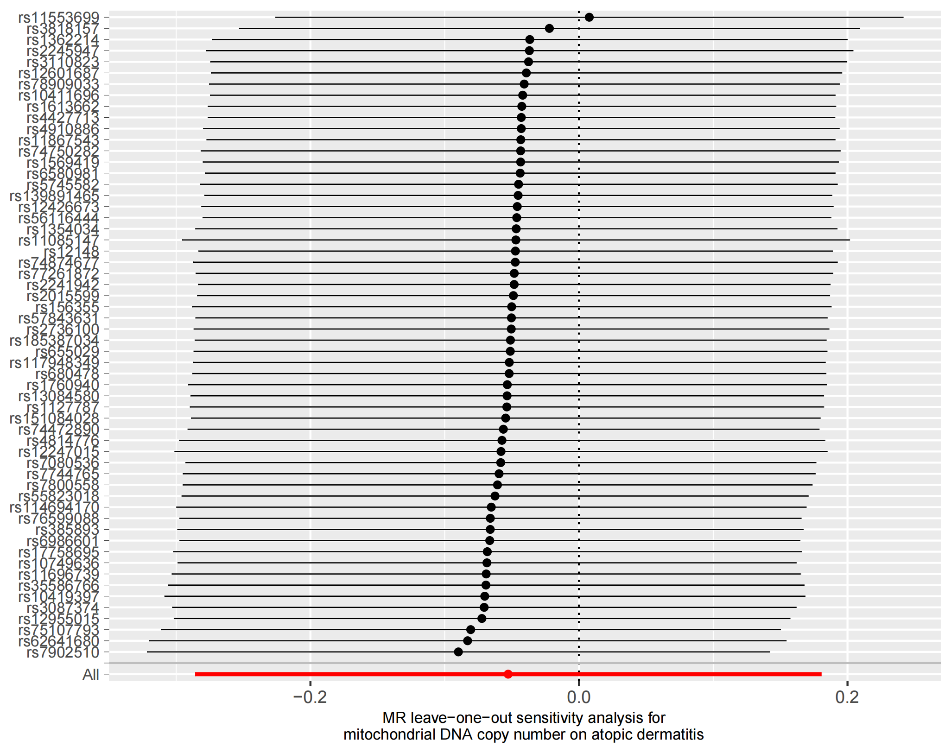

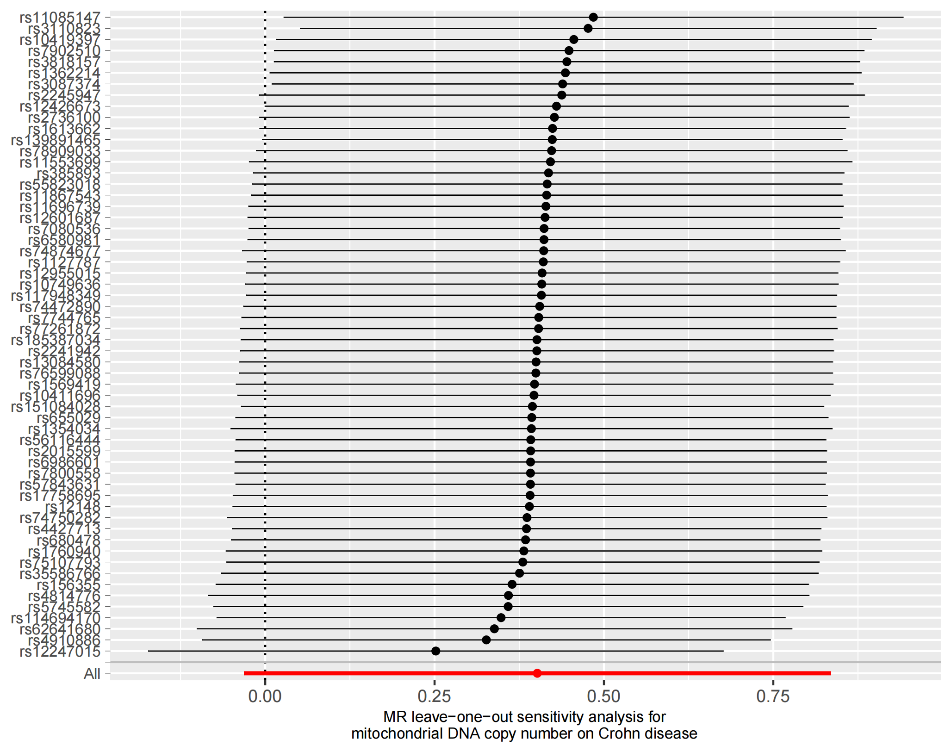


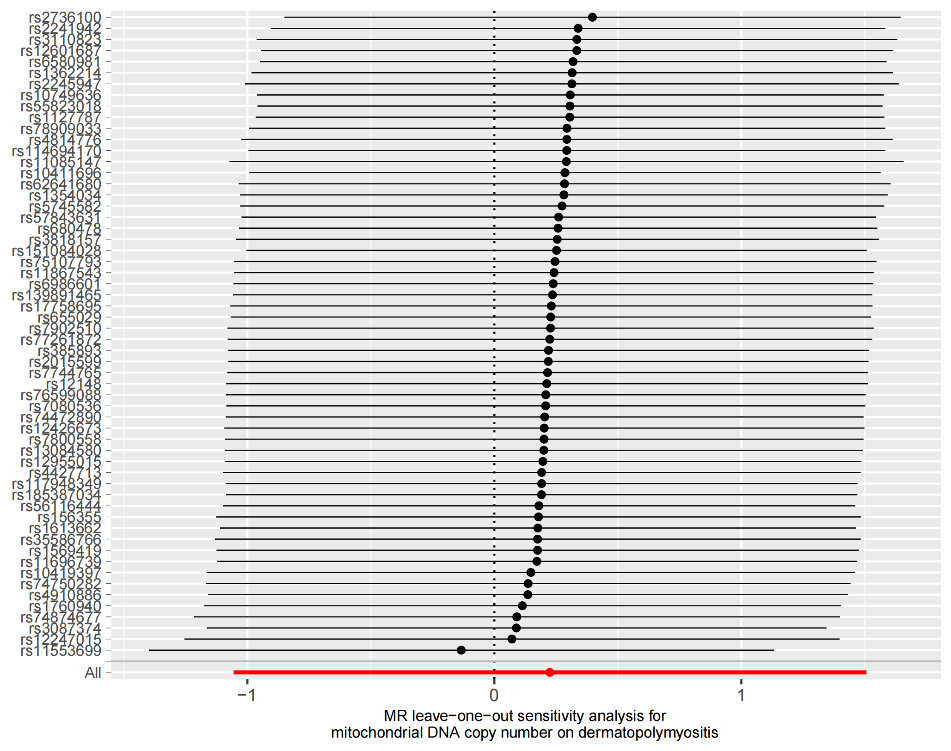

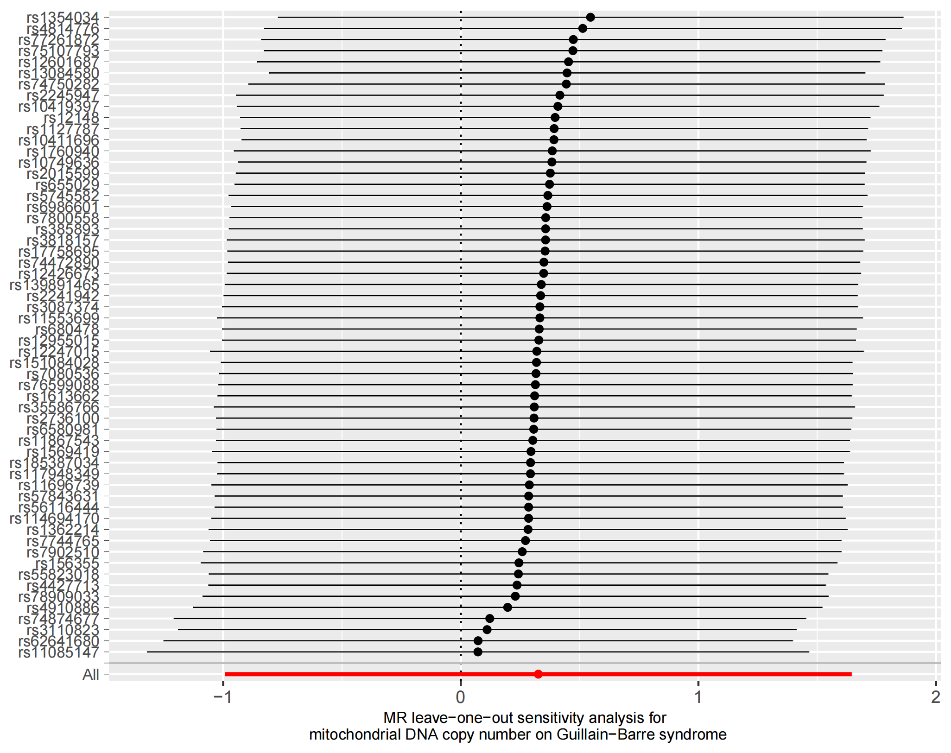


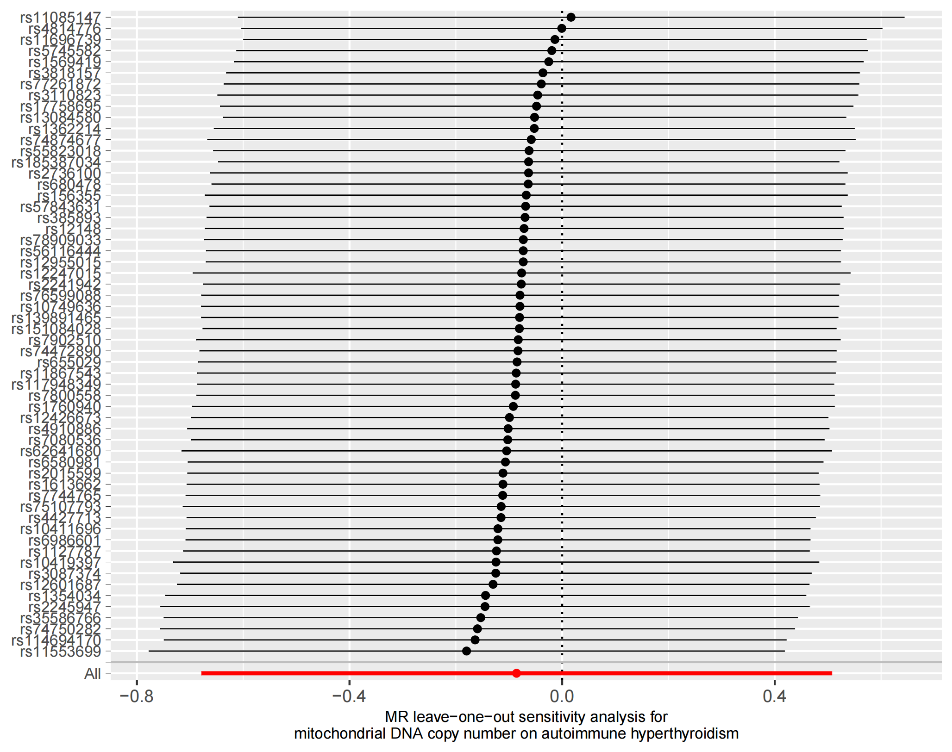

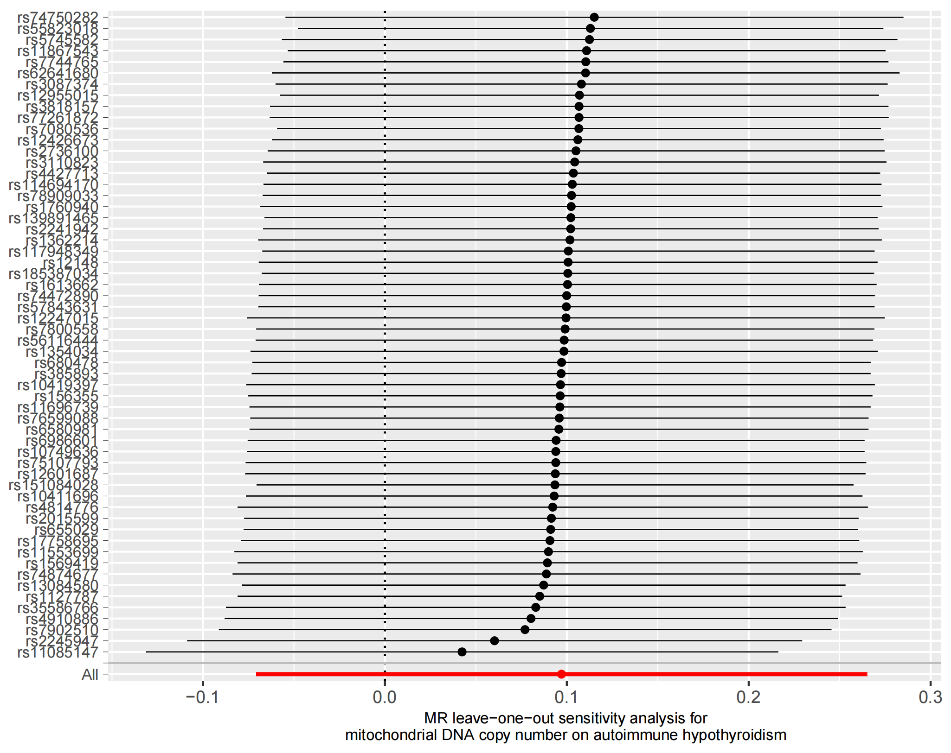


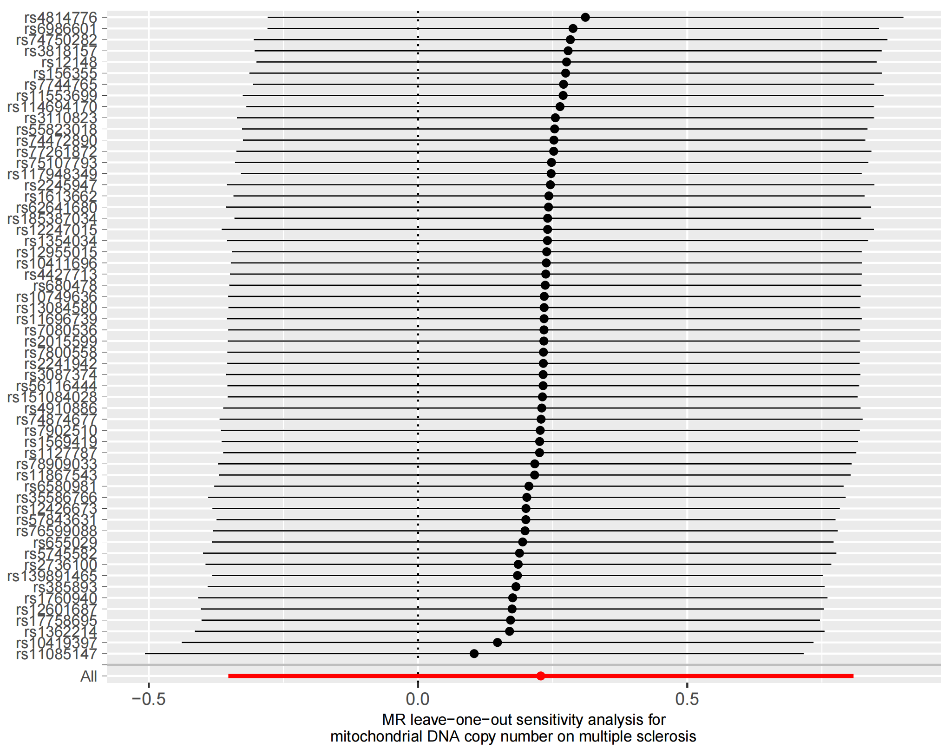

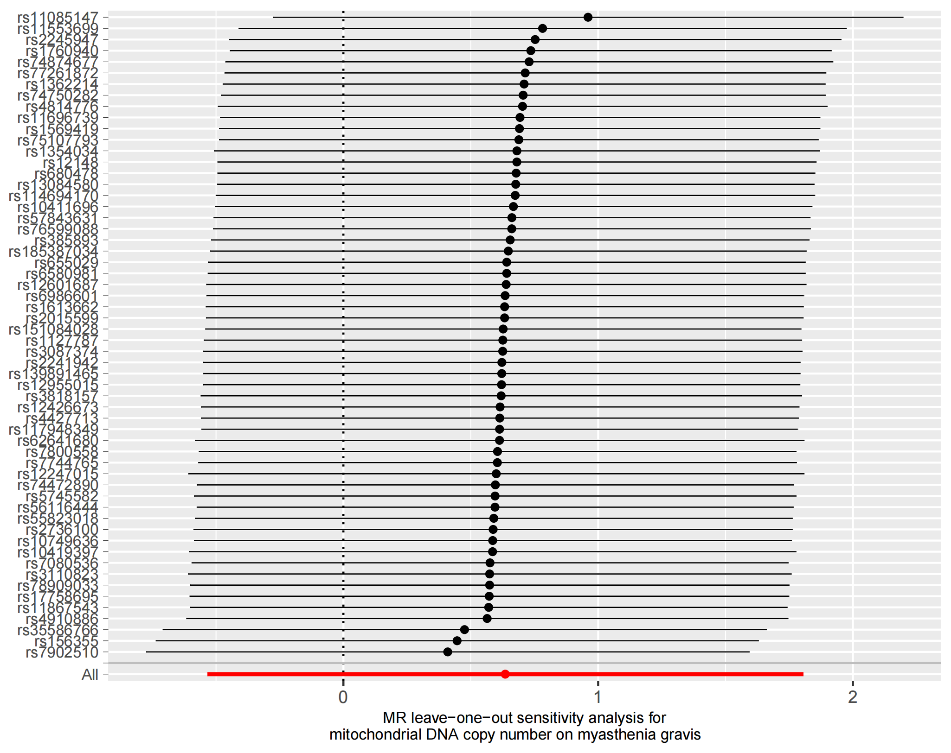


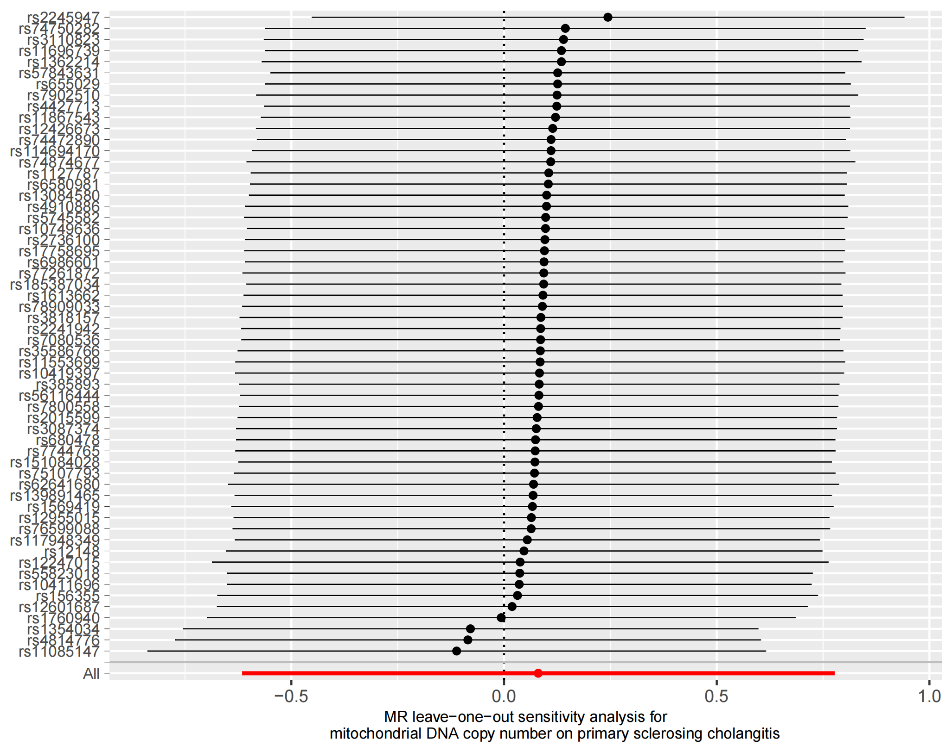

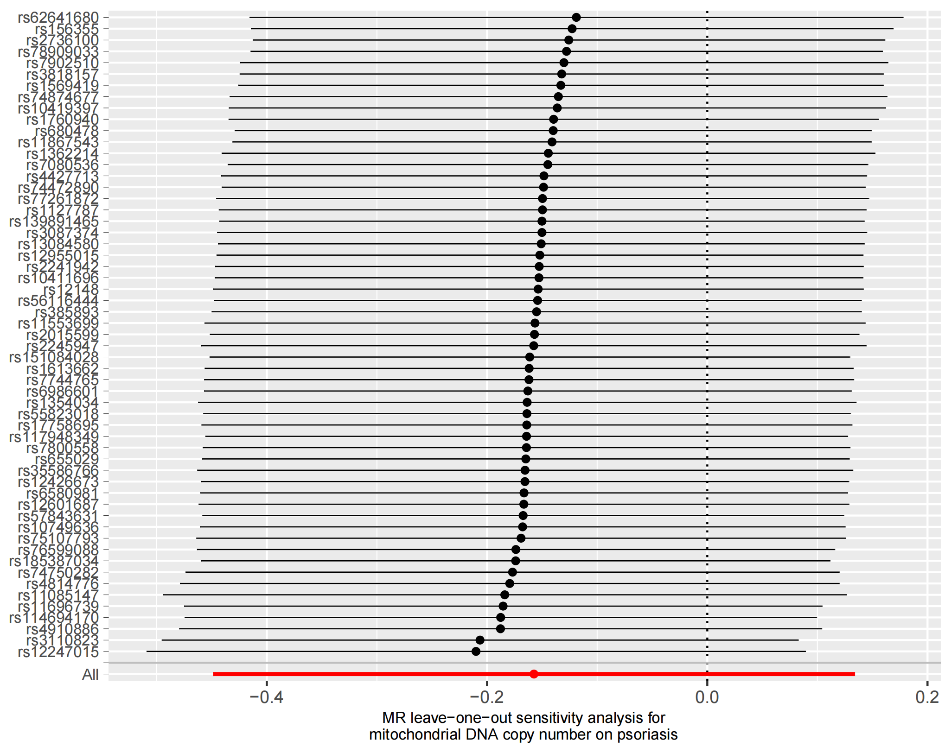


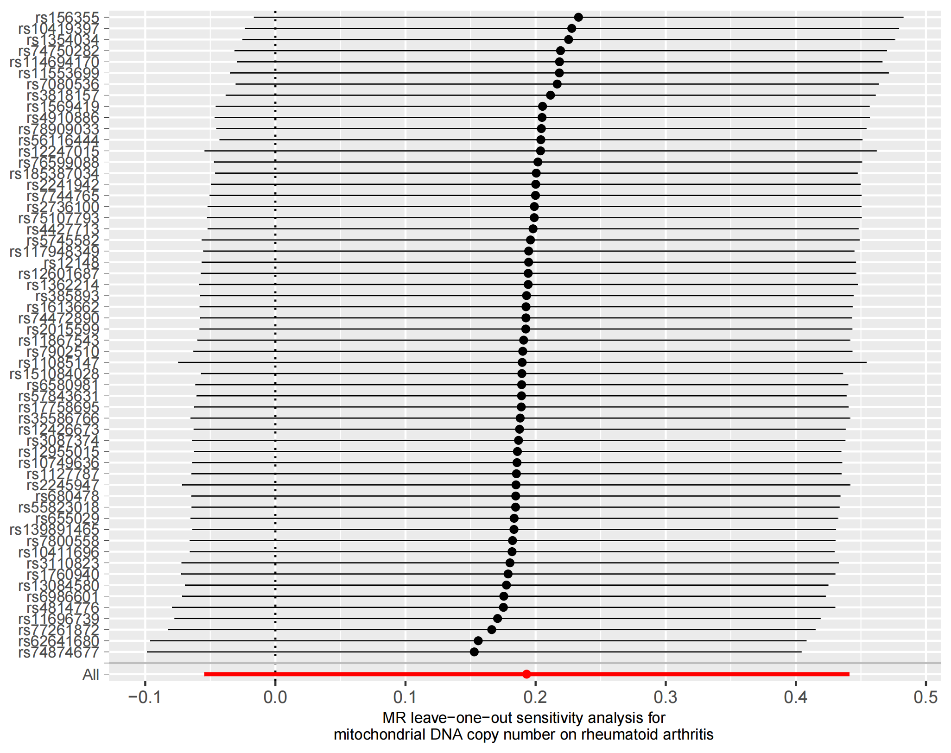

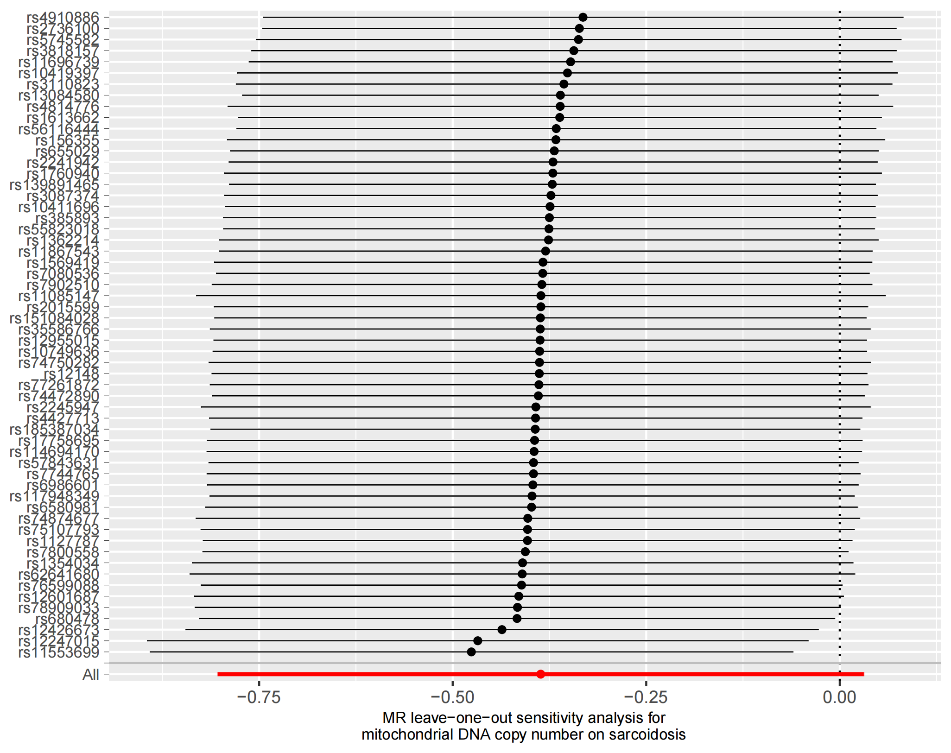


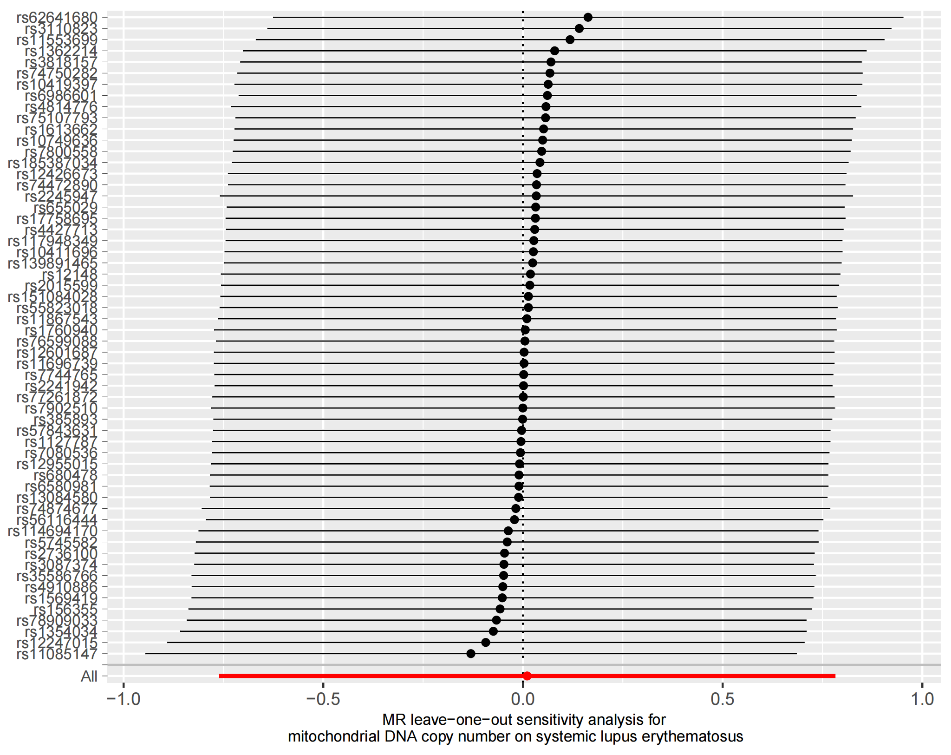

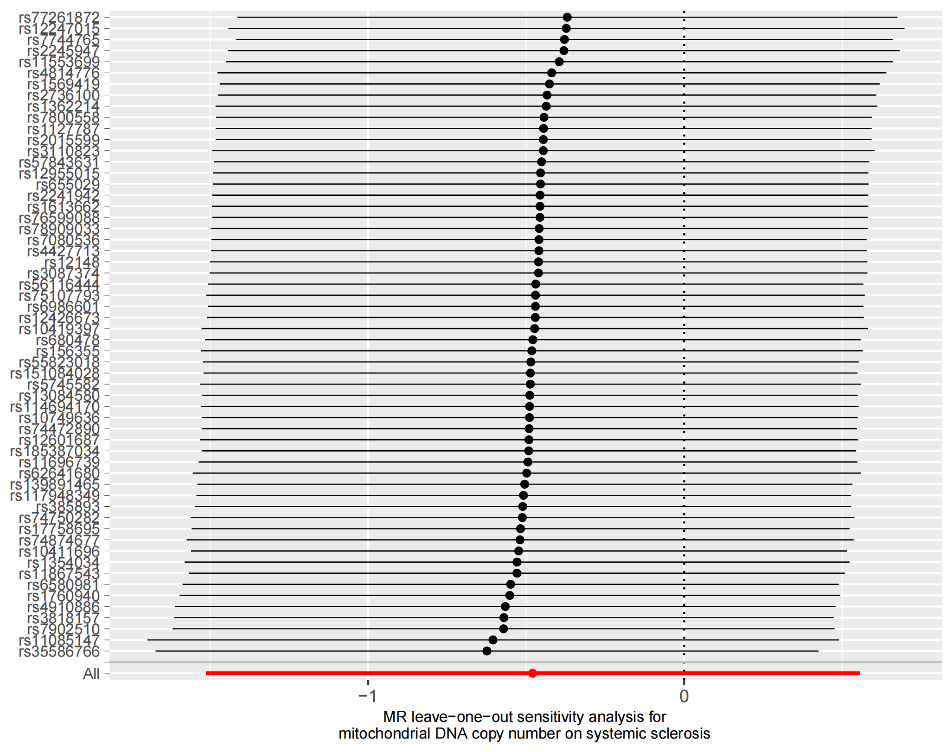


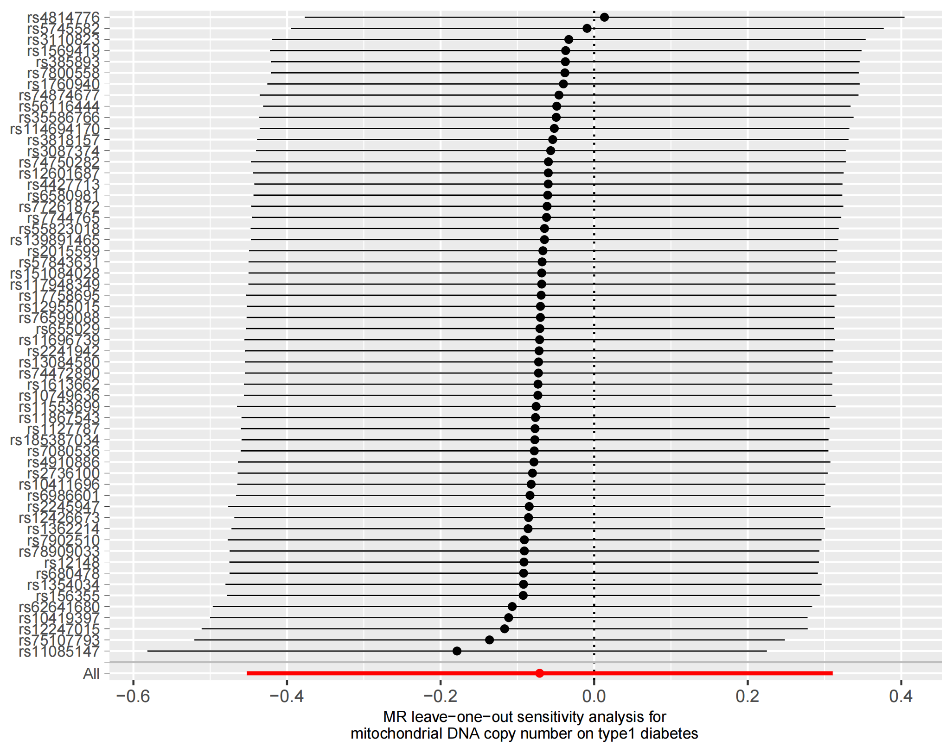

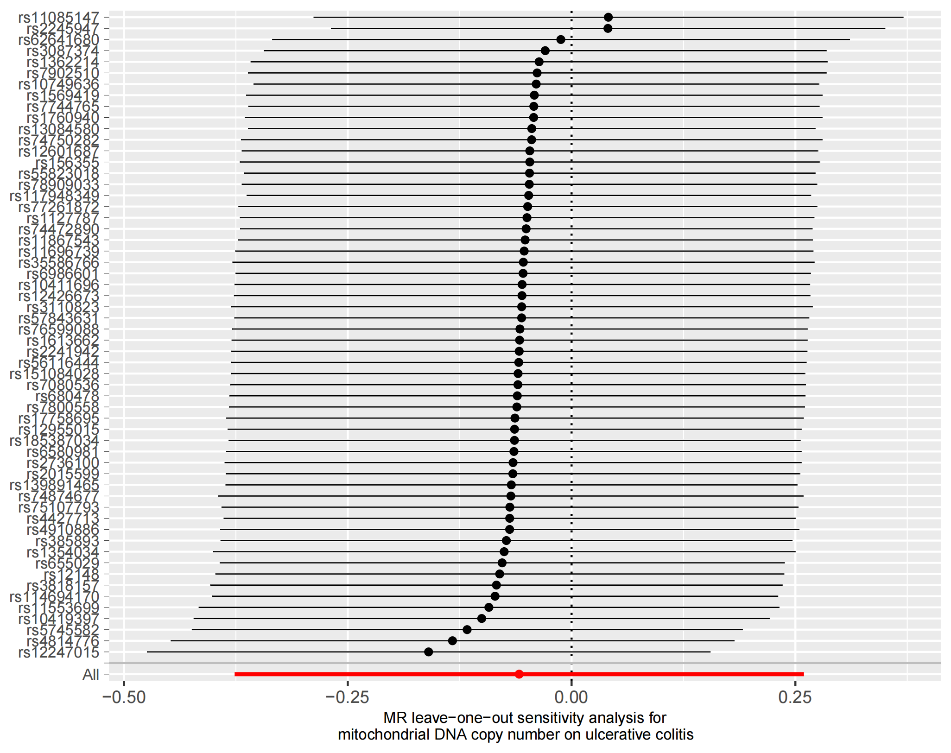


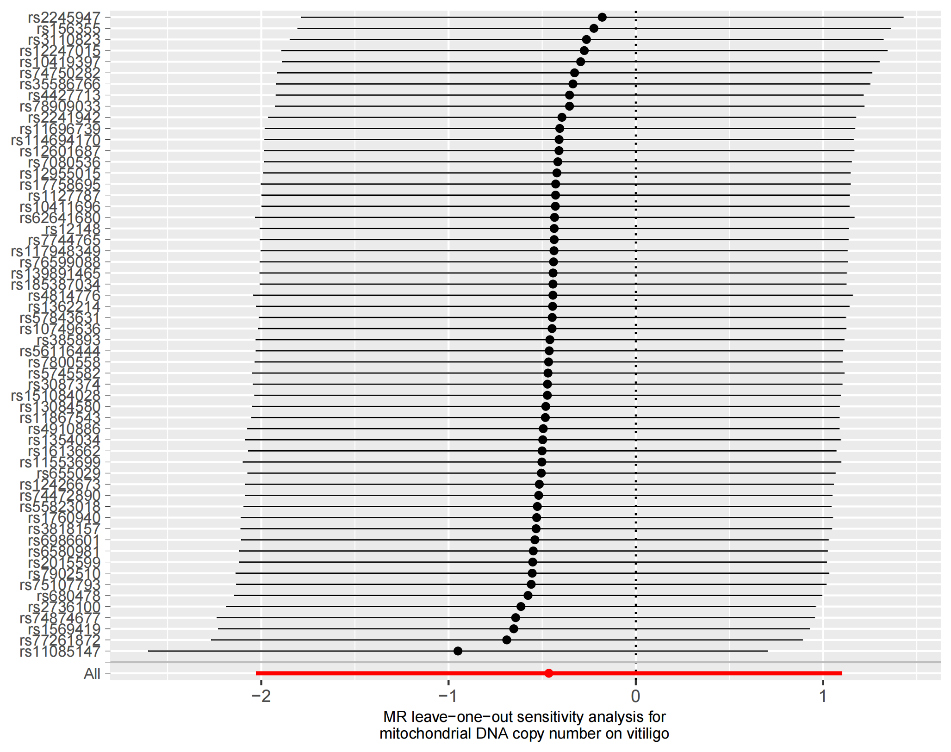


**Supplementary Figure 2.** Forest plots for the Mendelian randomization (MR) leave-one-out analysis based on IVs-2.


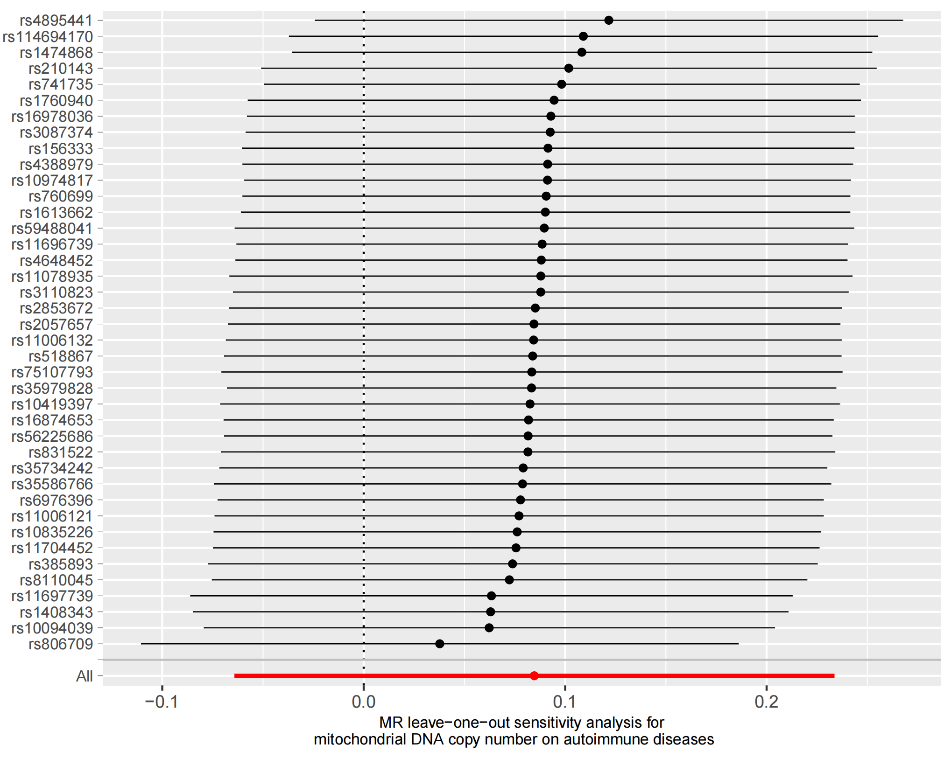

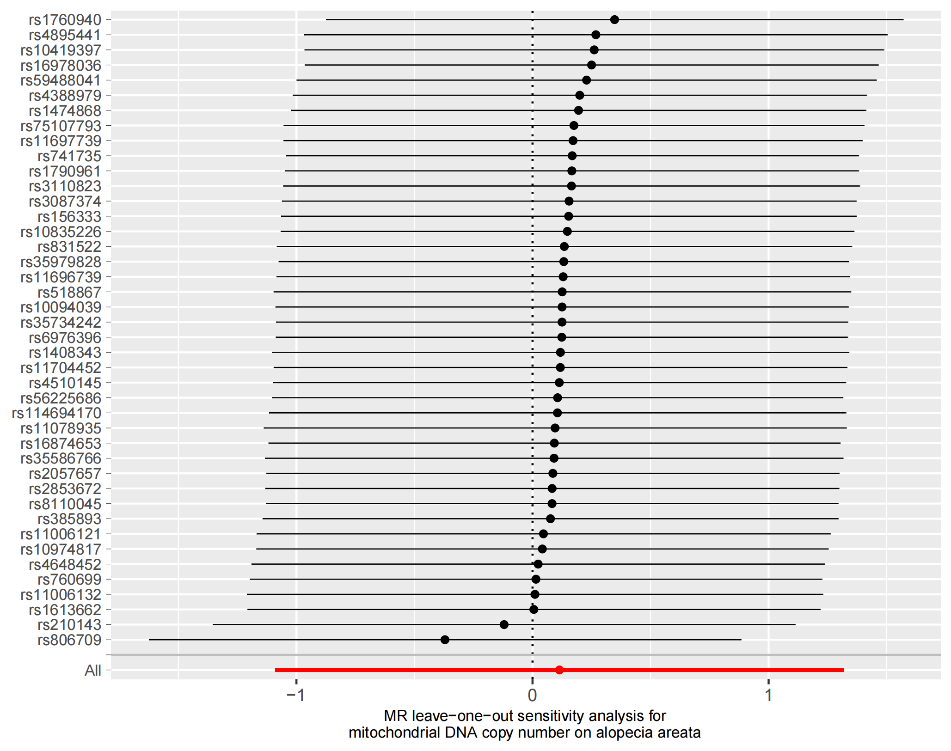


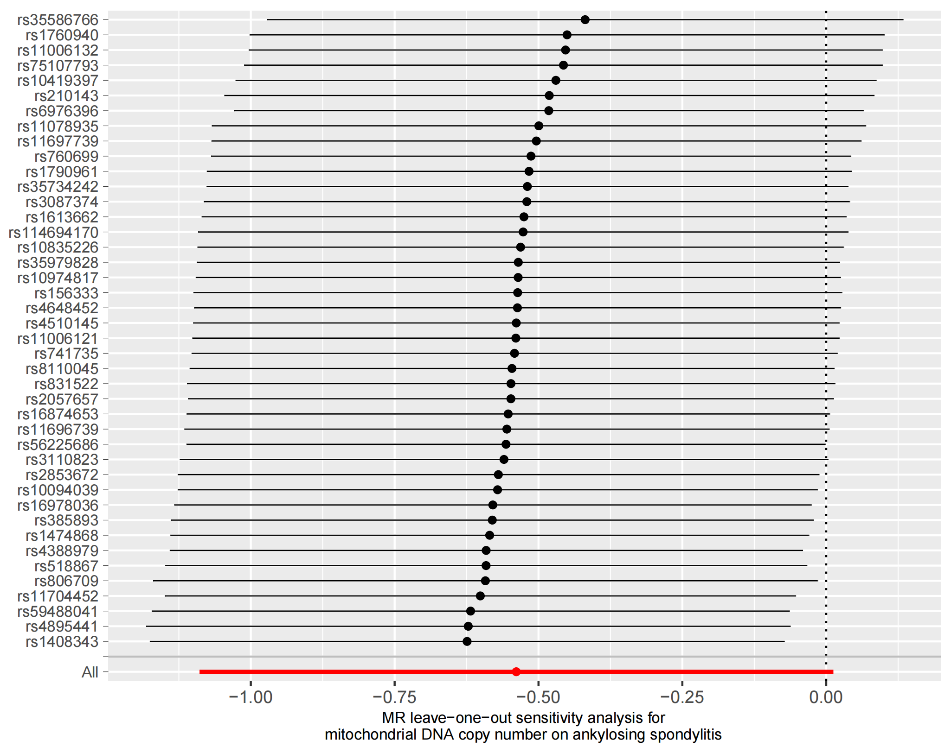

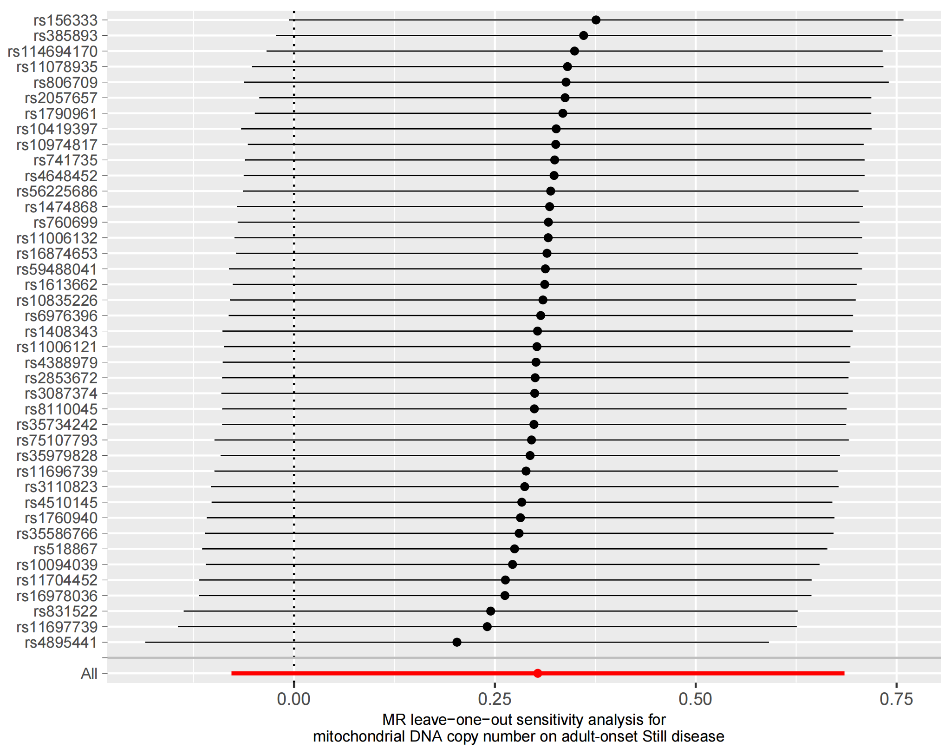


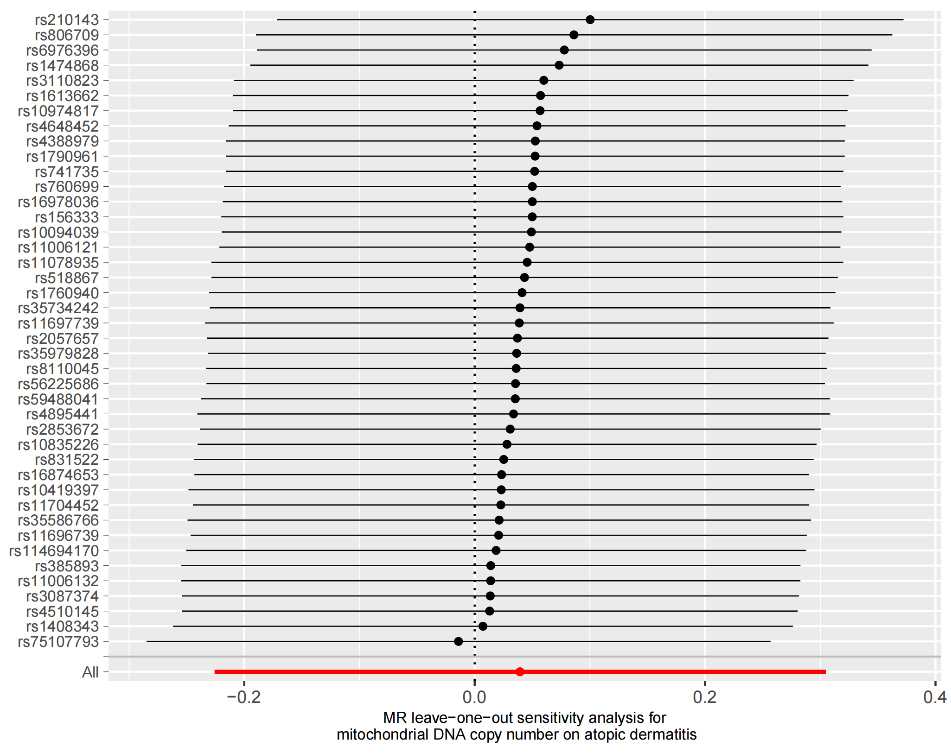

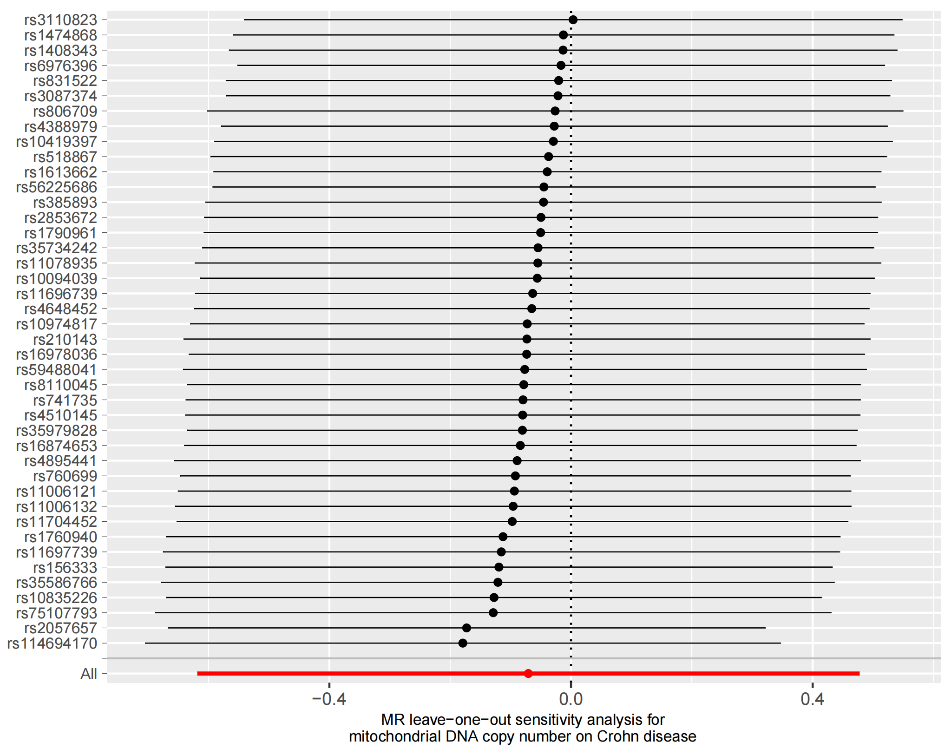


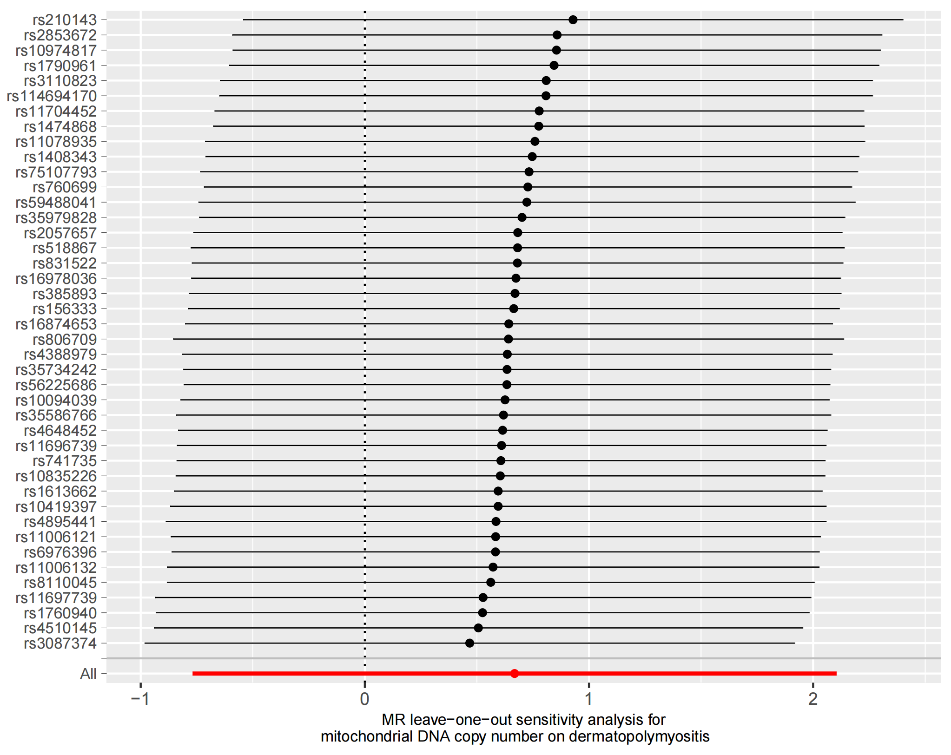

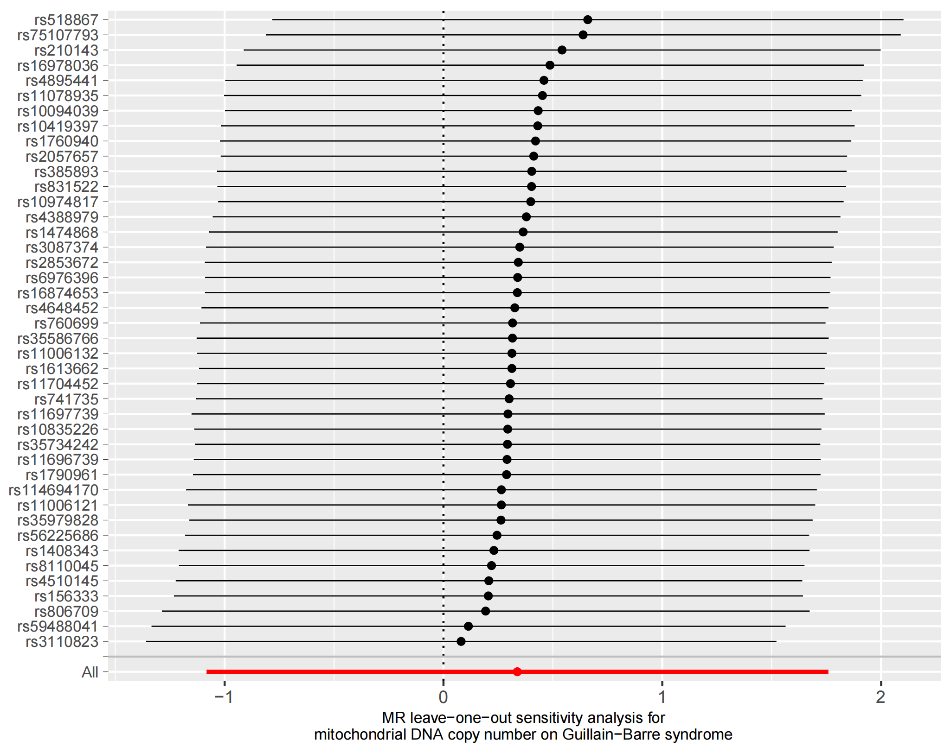


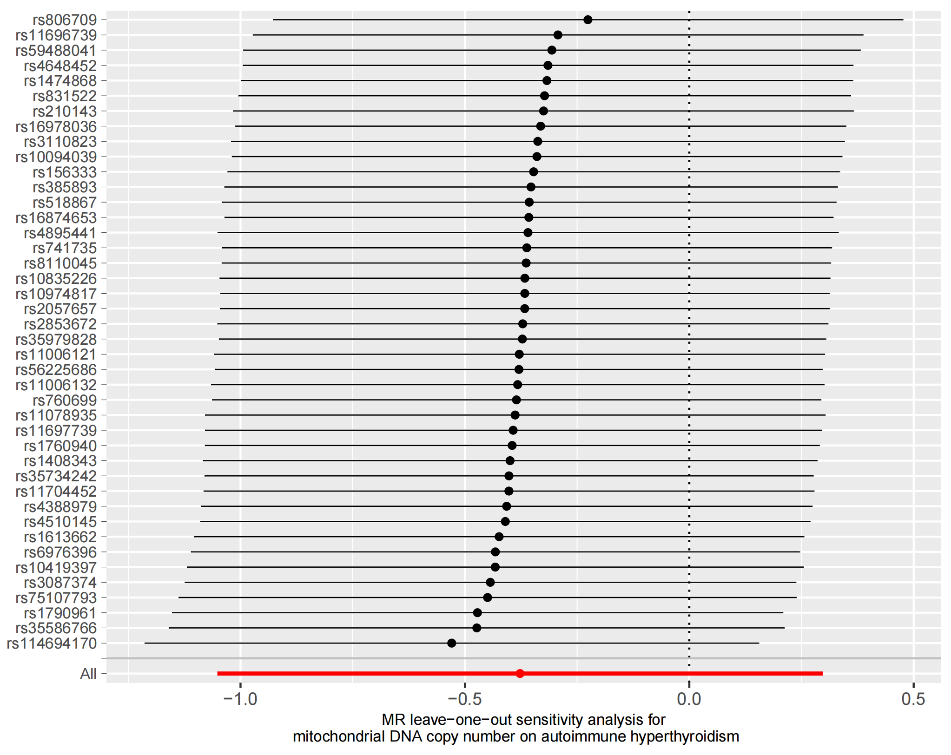

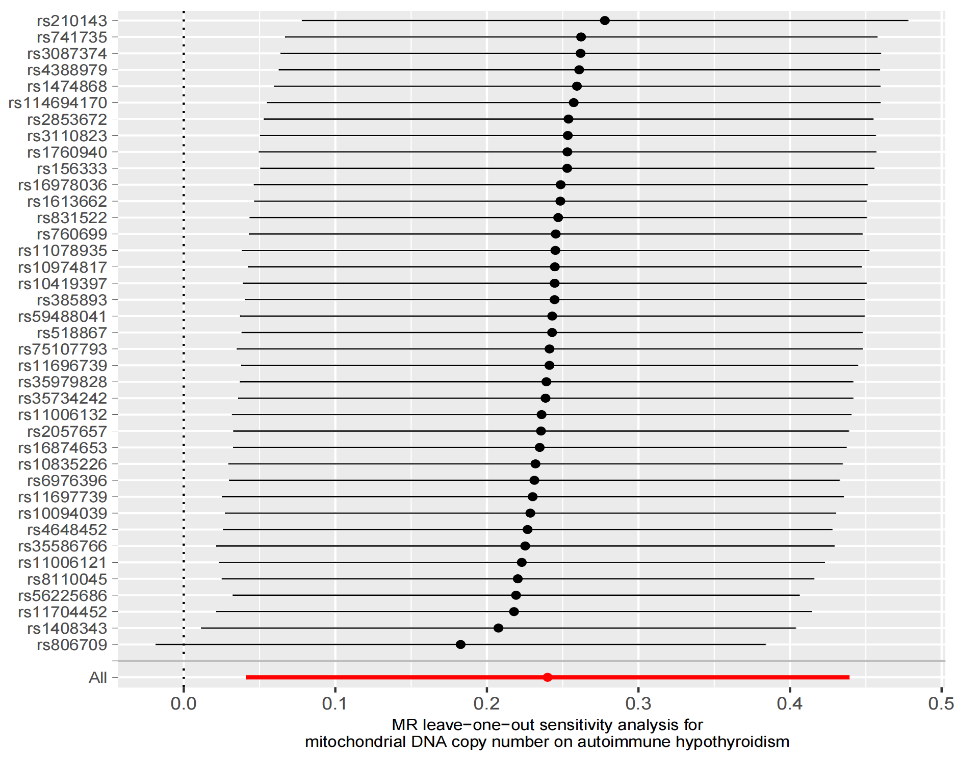


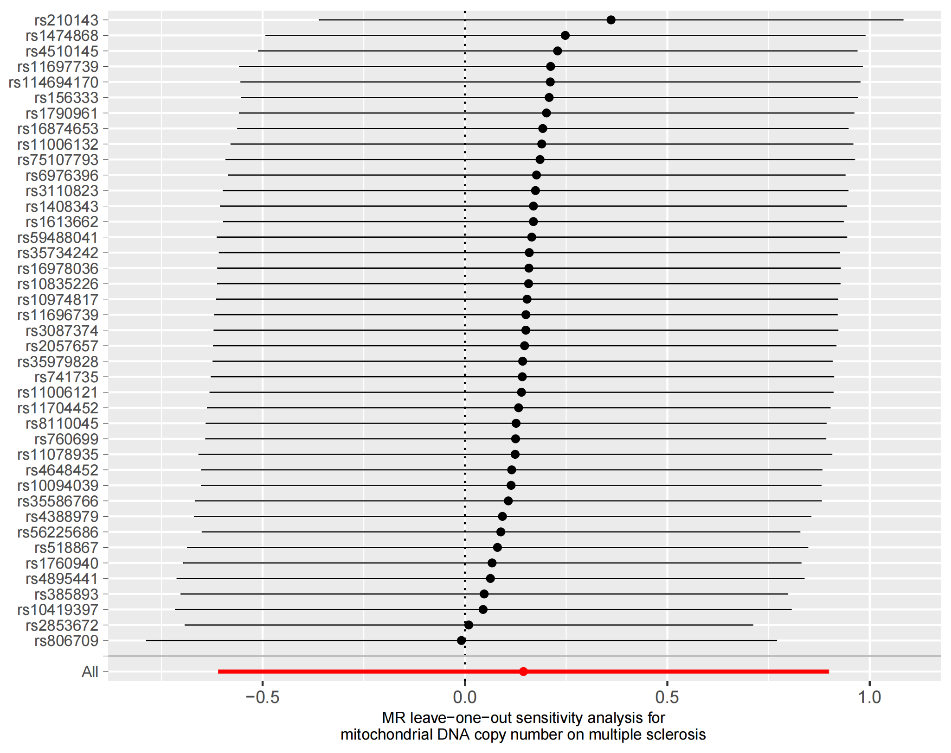

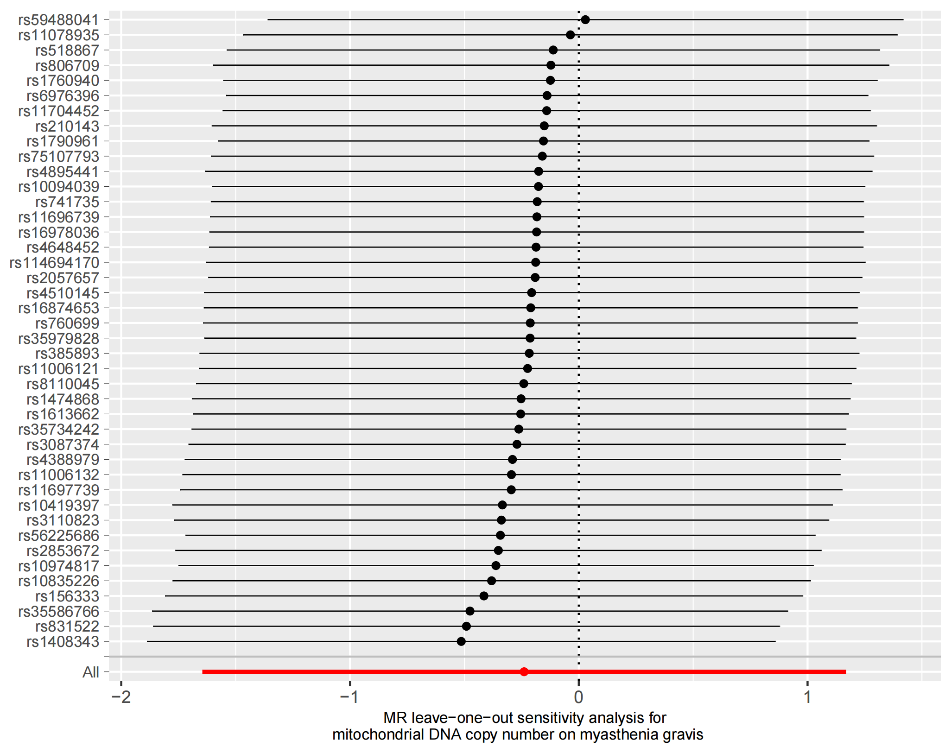


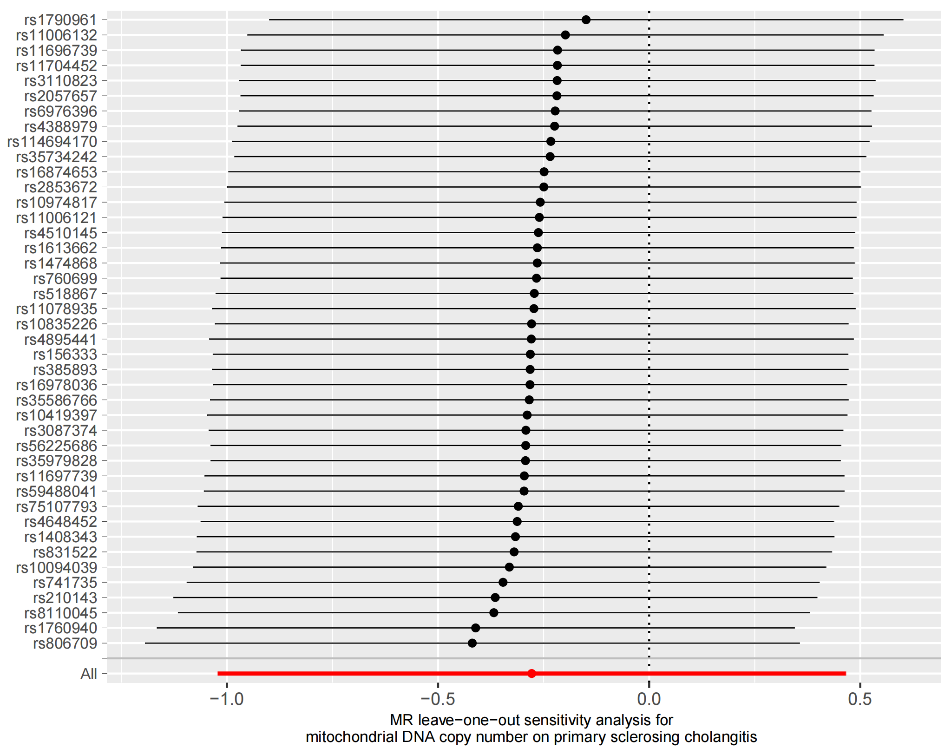

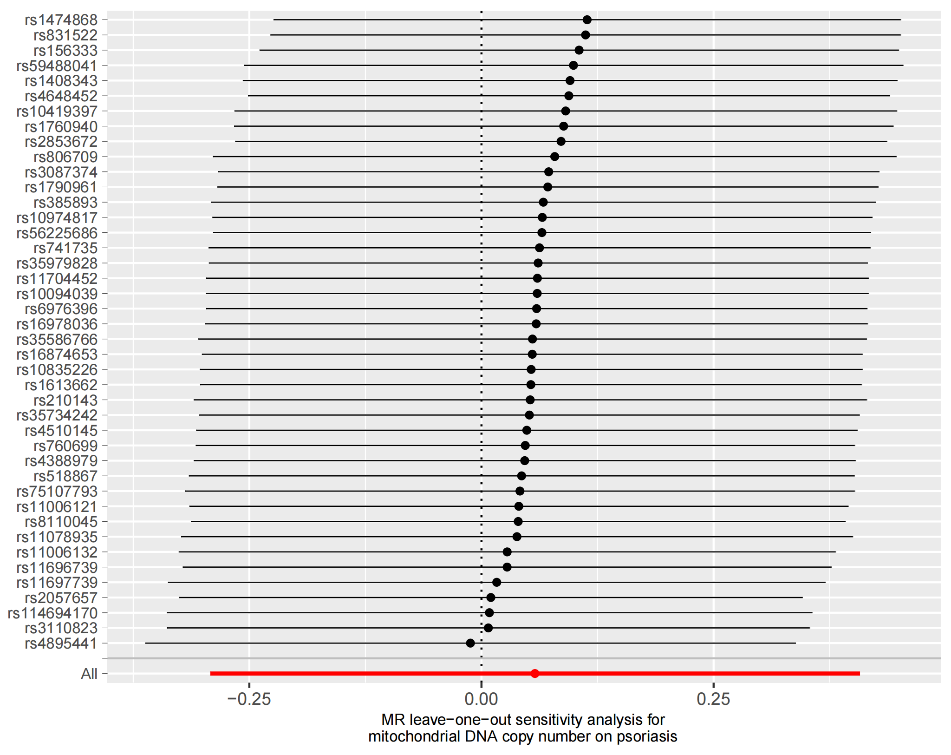


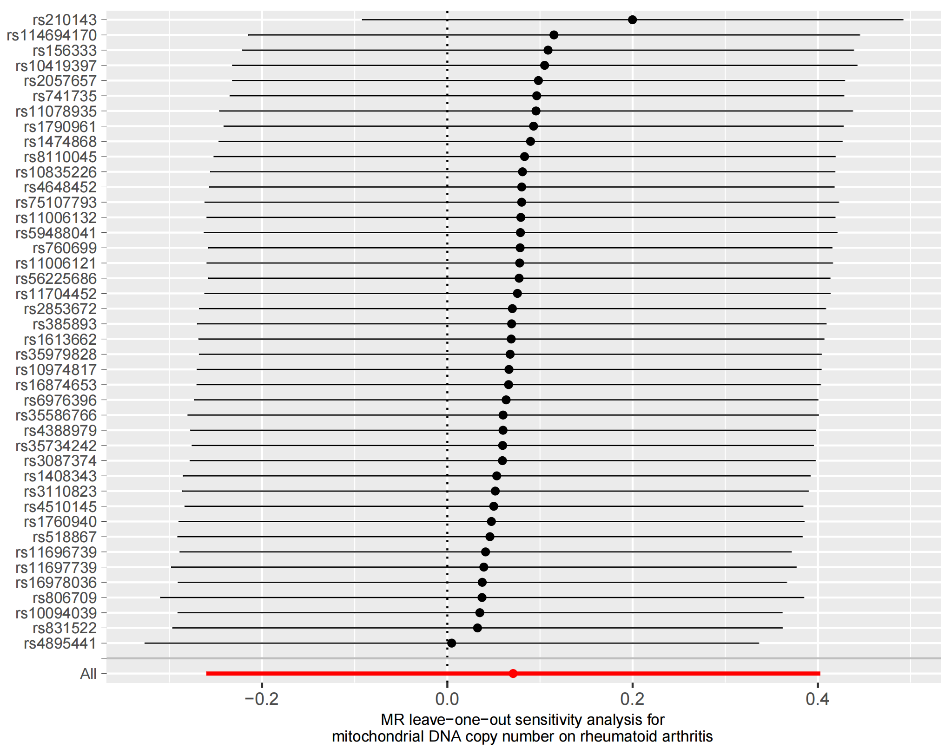

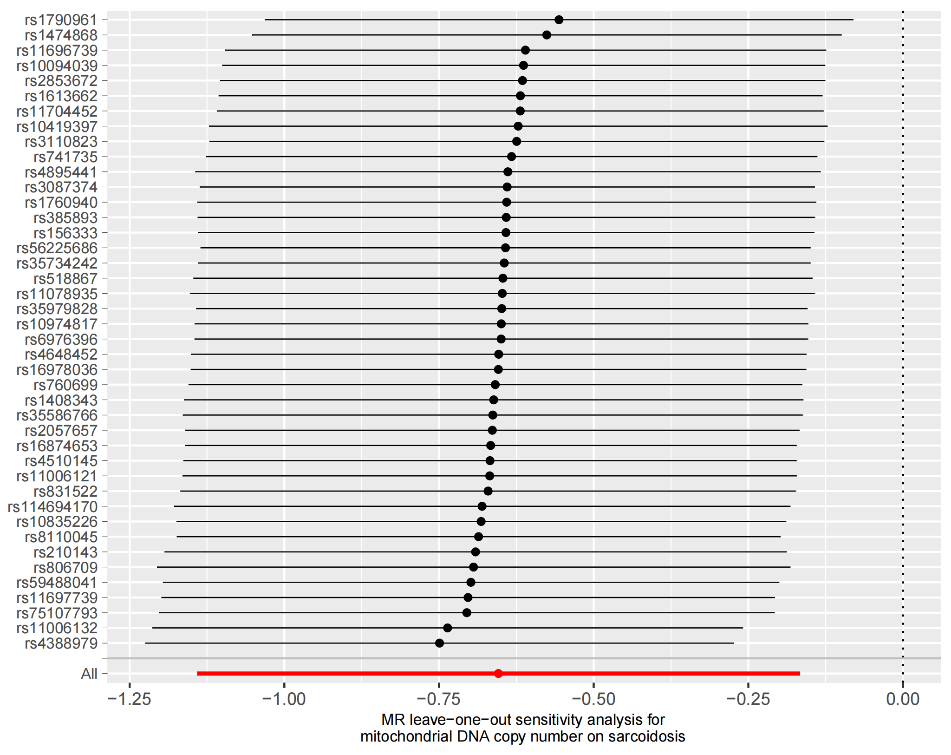


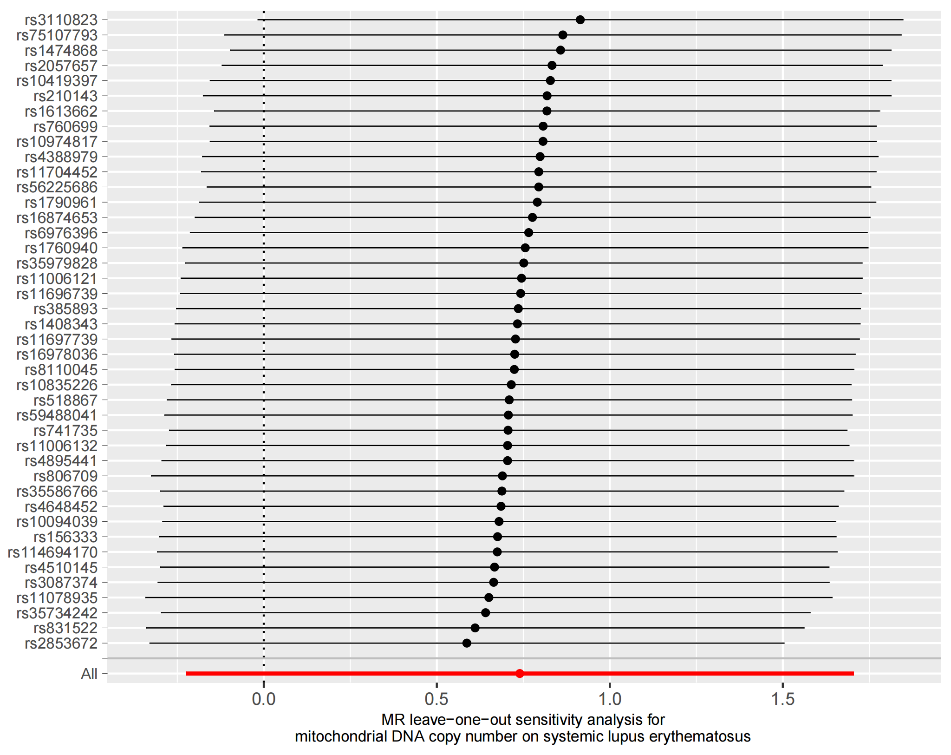

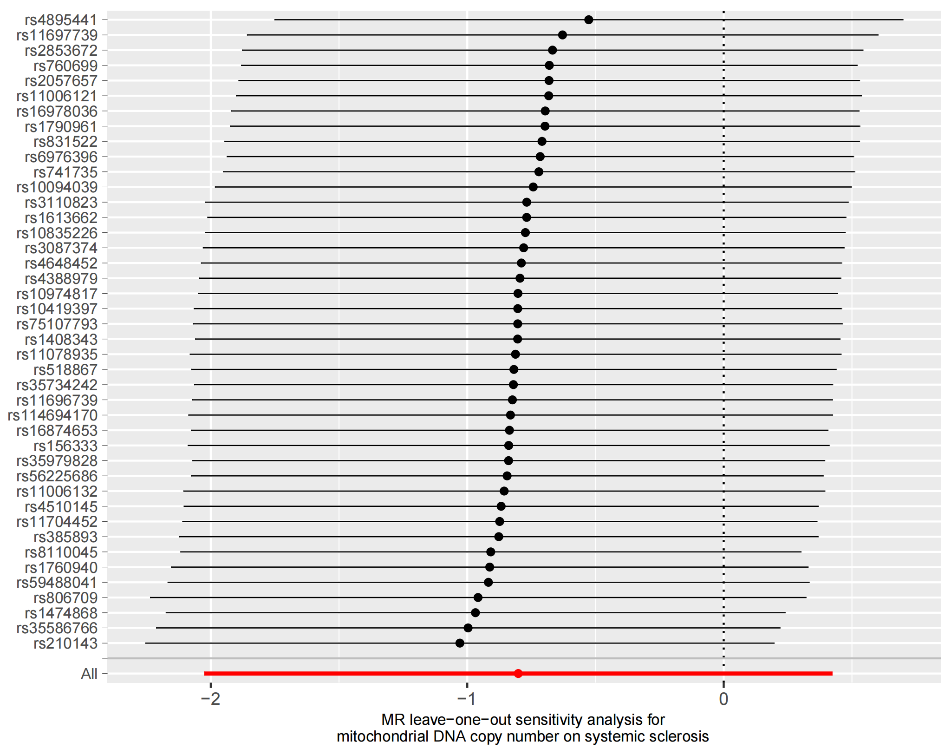


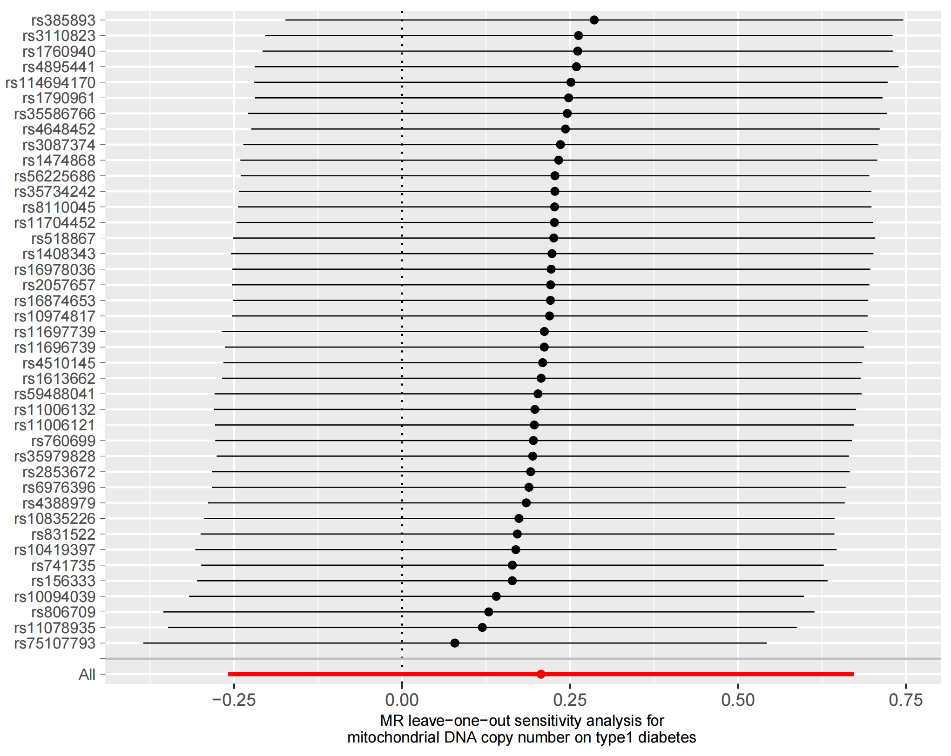

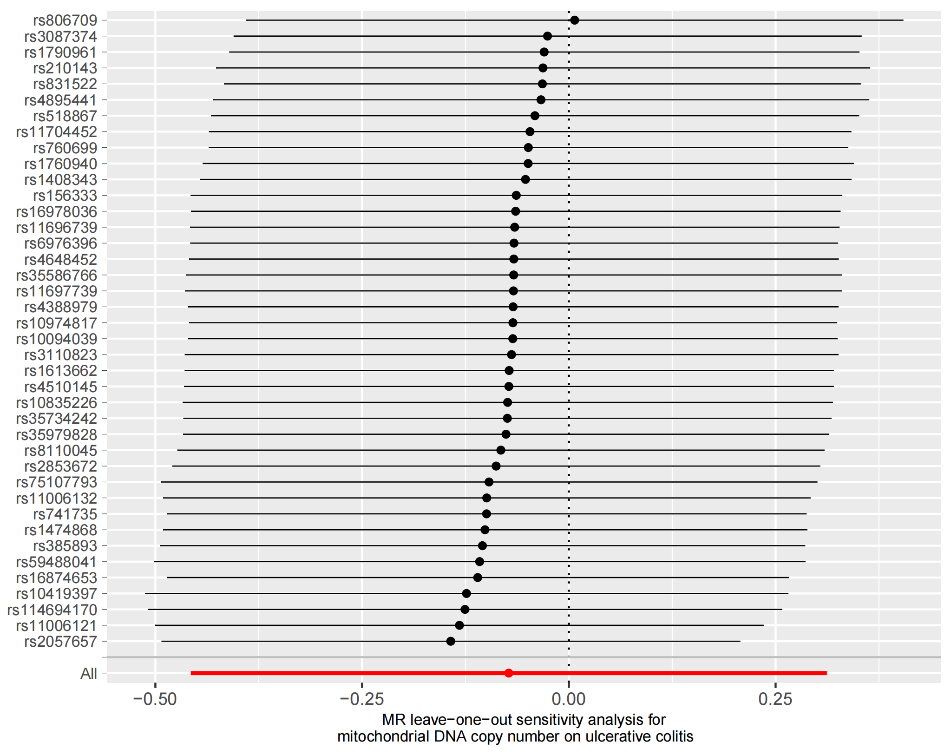


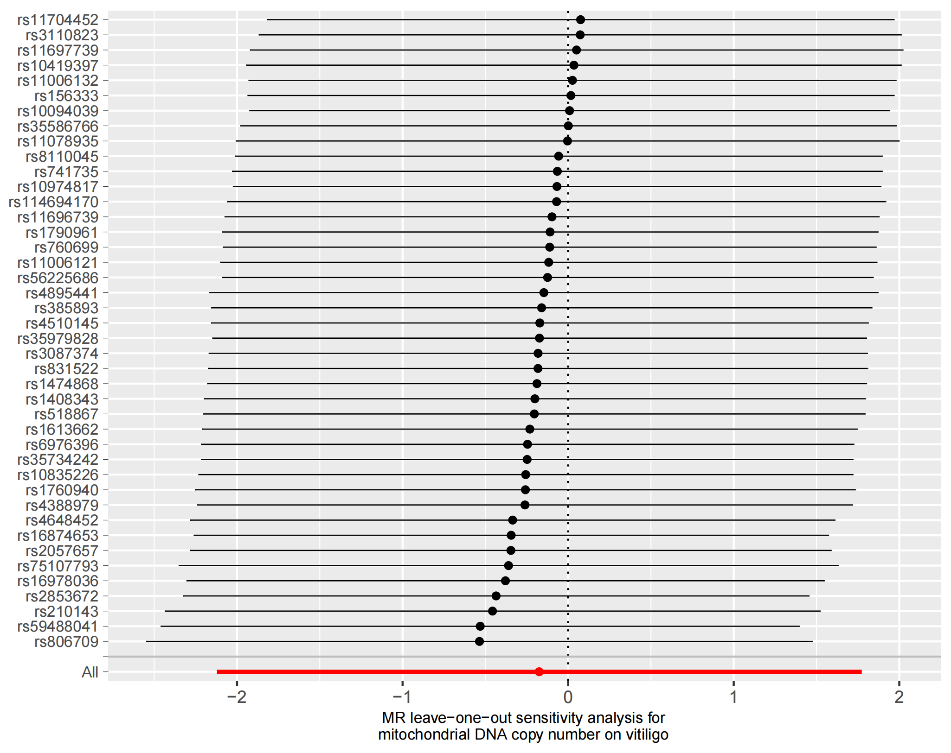


**Supplementary Figure 3.** Forest plots for the Mendelian randomization (MR) leave-one-out analysis based on IVs-3.
